# SIRT3 and GCN5L regulation of NADP+- and NADPH-driven reactions of mitochondrial isocitrate dehydrogenase IDH2

**DOI:** 10.1038/s41598-020-65351-z

**Published:** 2020-05-26

**Authors:** Katarína Smolková, Jitka Špačková, Klára Gotvaldová, Aleš Dvořák, Alena Křenková, Martin Hubálek, Blanka Holendová, Libor Vítek, Petr Ježek

**Affiliations:** 1 Laboratory of Mitochondrial Physiology, No.75, Institute of Physiology of the Czech Academy of Sciences (IPHYS CAS), Vídeňská 1083, 14220 Prague, Czech Republic; 2 Institute of Organic Chemistry and Biochemistry of the Czech Academy of Sciences (IOCB CAS), Prague, Czech Republic; 3 Institute of Medical Biochemistry and Laboratory Diagnostics, 1st Faculty of Medicine, Charles University, Prague, Czech Republic; 4 Present Address: Institute of Medical Biochemistry and Laboratory Diagnostics, 1st Faculty of Medicine, Charles University, Prague, Czech Republic

**Keywords:** Kinases, Mitochondria, Acetylation

## Abstract

Wild type mitochondrial isocitrate dehydrogenase (IDH2) was previously reported to produce oncometabolite 2-hydroxyglutarate (2HG). Besides, mitochondrial deacetylase SIRT3 has been shown to regulate the oxidative function of IDH2. However, regulation of 2HG formation by SIRT3-mediated deacetylation was not investigated yet. We aimed to study mitochondrial IDH2 function in response to acetylation and deacetylation, and focus specifically on 2HG production by IDH2. We used acetylation surrogate mutant of IDH2 K413Q and assayed enzyme kinetics of oxidative decarboxylation of isocitrate, 2HG production by the enzyme, and 2HG production in cells. The purified IDH2 K413Q exhibited lower oxidative reaction rates than IDH2 WT. 2HG production by IDH2 K413Q was largely diminished at the enzymatic and cellular level, and knockdown of SIRT3 also inhibited 2HG production by IDH2. Contrary, the expression of putative mitochondrial acetylase GCN5L likely does not target IDH2. Using mass spectroscopy, we further identified lysine residues within IDH2, which are the substrates of SIRT3. In summary, we demonstrate that 2HG levels arise from non-mutant IDH2 reductive function and decrease with increasing acetylation level. The newly identified lysine residues might apply in regulation of IDH2 function in response to metabolic perturbations occurring in cancer cells, such as glucose-free conditions.

## Introduction

Lysine acylation is a reversible post-translational modification representing one of the primary regulatory mechanisms in mitochondria, typically inhibition of protein function, and includes acetylation, malonylation, succinylation, glutarylation, *etc*.^1^. Mitochondrial lysine deacetylation is controlled by the sirtuin 3 (SIRT3), a mitochondrial ortholog of SIRT1, acting as the specific NAD^+^-dependent deacetylase. SIRT3-dependent lysine modification affects the ^N^ε-amino group of lysines, acetylation of which eliminates the positive charge. SIRT3 targets numerous mitochondrial enzymes, including mitochondrial respiratory chain subunits^2^, MnSOD^3^, and also morphology shaping proteins such as OPA1^4^. In general, SIRT3 activity promotes oxidative phosphorylation and catabolic metabolic pathways. Loss of SIRT3 has a pleiotropic effect in a number of diseases including cancer^5^; it is regarded as both a tumor suppressor protein^6,7^, and as a protein possibly stabilizing cancer survival^8^.

Mitochondrial isocitrate dehydrogenase 2 (IDH2), similarly to IDH3, catalyzes the oxidative decarboxylation of isocitrate (IC) into 2-oxoglutarate (2OG) and CO_2_, albeit using cofactor NADP^+^. IDH2 is a homodimer containing two binding sites for NADPH, IC, and Mg^2+^, essential for the reaction. The specific metabolism of malignant cells related to glutaminolysis also involves the reductive carboxylation (RC) reaction of IDH2, supporting the citrate export from mitochondria followed by lipid synthesis^9^. On the other hand, heterozygous gain-of-function IDH2 mutations, such as those found in grade 3 to 4 gliomas, promote CO_2_-independent reduction of 2OG into 2-hydroxyglutarate (2HG)^10^. However, we and others have previously demonstrated that wild-type IDH2 (IDH2 WT) produces 2HG in breast cancer cells; *i.e*., in HTB-126/Hs578T cells, 2HG is readily formed in the absence of known IDH2 mutations, and IDH2 WT was identified to be responsible for its substantiate part^11,12^.

There are discrepancies regarding the effect of acetylation on IDH2 activity. The original report^13^ states that the IDH2 activity drops after acetylation and rises after SIRT3-mediated deacetylation with concomitant improvement of mitochondrial redox status, including elevated glutathione levels. These findings were supported by the report of induced IDH2 dimerization in response to deacetylation, while ablation of *SIRT*3 resulted in the decrease of IDH2 dimerization and activity; similarly, mimicking acetylation of lysine 413 by the IDH2 K413Q mutant also abolished dimerization^14^. In contrast, the acetylated IDH2 was found to exhibit higher activity than the non-acetylated IDH2^15^. These disagreements call for further studies.

Acylation of mitochondrial proteins occurs both non-enzymatically and as enzyme-catalyzed. Non-enzymatic acylation arises due to the alkaline pH in the mitochondrial matrix^16^ and a relatively high concentration of acetyl-CoA and succinyl-CoA (0.1–1.5 mM and 0.6 mM, respectively)^1,16^. Additionally, lysine acetylation was also indicated in hepatocytes, as promoted by mitochondrial fatty acid β-oxidation of palmitate in a low glucose medium^17^. On the other hand, acetyl-CoA acetyltransferase 1 (ACAT1) has been reported to acetylate the pyruvate dehydrogenase^18^. Concerning the enzyme-catalyzed acetylation, lysine acetyl-transferase termed General Control of Amino Acid Synthesis 5-like 1 (GCN5L1, also termed BLOS1, BLOC1S1) has been identified based on homology with the nuclear acetyltransferase GCN5^19^. Besides the peroxisomes, GCN5L1 also localizes to mitochondria and acetylates various substrates *in vitro*, including the ATP-synthase subunit α^19^. GCN5L ablation induced PGC-1α and consequent mitochondrial biogenesis, and, simultaneously, concurrent mitophagy due to lysosomal biogenesis by activation of the transcription factor recognizing E-box sequences^20,21^. In the liver, the loss of GCN5L results in the reactive oxygen species- (ROS) dependent activation of ERK, down-regulation of FoxO1, and inhibition of gluconeogenesis^22^. Moreover, GCN5L1 expression increased in response to a high-fat diet in the heart muscle, where GCN5L1 acetylation attenuates the activity of short-chain and long-chain acyl-CoA dehydrogenase and fatty acid β-oxidation^23^. These data provide evidence for a functional role of GCN5L in mitochondria and suggest yet another link for acetylation-deacetylation dynamics in response to nutrient availability.

In this study, we documented inhibition of 2HG production in response to acetylation and 2HG elevations at deacetylation. Using the acetylation surrogate mutant of IDH2, K413Q, overexpression of SIRT3 vs. GCN5L, or by SIRT3 silencing, we studied the resulting changes in the IDH2 kinetics of NADP^+^-driven reaction and 2HG-forming reduction of 2OG. Confirming the profound inhibitory role of IDH2 acetylation, we demonstrate that acetylation of IDH2 attenuates also the formation of 2HG by IDH2. Moreover, we identify different prospective sites of lysine deacetylation by SIRT3 within the IDH2 sequence.

## Results

### Both oxidative and reductive function of IDH2 are diminished by acetylation surrogate K413Q

To evaluate the role of acetylation for the IDH2 activity, we revisited the published data on the K413Q mutant of IDH2 (IDH2 K413Q), which represents an acetylation surrogate. Lysine 413 of IDH2 was identified as a plausible target of SIRT3-mediated deacetylation that regulates the function of IDH2^13^. The IDH2 K413Q mutant was reported to have reduced oxidative activity. For measuring of enzyme kinetics, IDH2 WT and K413Q enzymes were purified using Flag-tag affinity column. Purified enzymes were subjected to circular dichroism (CD) spectrometry^24^ and thermal stability measurement in order to determine whether such point mutation affects the secondary structure of IDH2 (Supplementary Fig. S1 A, B). CD spectra showed that IDH2 WT and K413Q proteins have similar numbers of secondary structures, and melting temperatures of both protein variants were 36.5 °C and 36.8 °C, respectively. So, mutation K413Q likely does not cause changes in protein´s structural stability.

**Figure 1:**
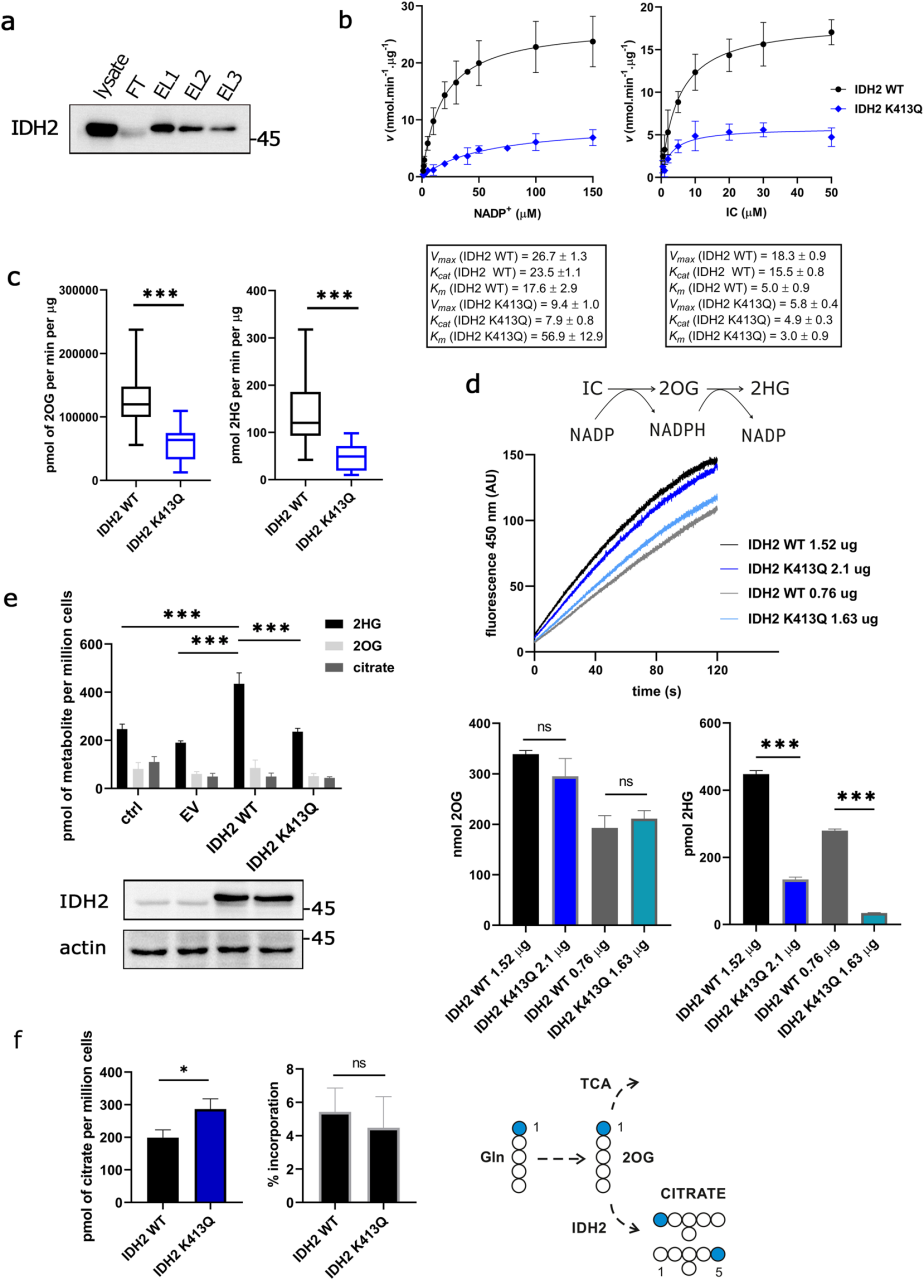
IDH2 reaction of IDH2 WT and IDH2 K413Q. **(a)** Western blot of the exemplar purification of IDH2; whole cell lysate (*lysate*), flow-through (*FT*) indicating that significant pool of IDH2-Flag is attached to the resin; three sequential steps of elution by Flag-peptide (*EL1*, *EL2*, *EL3*). **(b)** Michaelis-Menten plots for NADP^+^-driven IDH2-mediated NADPH formation as dependent on concentrations of NADP^+^ (left) and IC (right). Box contains values of *V*_*max*_ and *K*_*m*_. Unit of *K*_*cat*_ for IDH2 WT/K413Q is *s*^*−1*^. Data are expressed as mean ± s.e.m. **(c)** GC-MS evaluation of reaction products after 120 s of a purified enzyme in the reaction mixture, 2OG (left) and 2HG (right). N > 10, ***p < 0.0001 calculated using Unpaired t-test with Welch´s correction. **(d**) Reaction schema depicting 2HG formation in the *in-vitro* reaction; reactions of isolated IDH2 WT and K413Q as recorded by NADPH formation depicting similar reaction rates; underneath, 2OG and 2HG evaluation of the actual reaction products corresponding to presented reaction rates. N = 3, ***p < 0.0001 calculated using One Way ANOVA Tukey´s multiple comparisons test. **(e)** GC-MS evaluation of metabolites 2HG, 2OG and citrate extracted from the 293LTV cells transfected with no vector (“ctrl”), with the empty vector (“EV”), with vector encoding wild-type IDH2 (“IDH2 WT”) or K413Q mutant (“IDH2 K413Q”) with corresponding western blots. N = 3, ***p < 0.001 (p = 0.0001, p < 0.0001, p < 0.0001, respectively) calculated using One Way ANOVA Tukey’s multiple comparisons test. **(f)** Citrate levels in SHSY5Y expressing IDH2 WT and K413Q, respectively (left), and % incorporation calculated as M+0 and M+1 ratio of the respective ion form (middle), and the experimental scheme depicting ^13^C labeling of citrate from 1−^13^C-glutamine. N = 3, *p < 0.05 (p = 0.0186) calculated by Unpaired t-test.

Next, we evaluated the kinetics of the purified IDH2 WT (Fig. 1a) enzyme and compared it to the IDH2 K413Q. A feasible assay of IDH2 activity includes IC and NADP^+^ as the initial substrates (Supplementary Fig. S1 C). This gives rise to 2OG and NADPH by decarboxylation. The reaction kinetics, *i.e*. NADP^+^-driven NADPH production, was estimated as reaction rates at different NADP^+^ or IC concentrations (Fig. 1b). The saturated NADPH production was indeed much lower (<33%) for the K413Q IDH2 mutant (Fig. 1b; Supplementary Fig. S1). This was reflected by Michaelis-Menten and Eadie-Hofstee plots, where the K413Q IDH2 mutant exhibited substantially decreased *V*_*max*_ values (Fig. 1b; Supplementary Fig. S1). The apparent affinity for NADP^+^ of the IDH2 K413Q protein decreased, whereas for IC, the apparent affinity remained unchanged (Fig. 1b).

According to a reaction scheme (Fig. 1d), 2HG can be produced *in vitro* from 2OG following decarboxylation of IC and consuming NADPH. To estimate the yield of metabolites produced by IDH2 WT and K413Q, we evaluated 2OG and 2HG in the reaction mixture after 120 s of incubation using GC-MS. The 2OG content at 120 s was diminished in IDH2 K413Q compared to IDH2 WT, along with 2HG (Fig. 1c). However, it is intriguing to consider that the obtained 2HG production of IDH2 K413Q samples would be lower because of proportionally lower 2OG production (which is a substrate for 2HG-forming reaction), as inferred from results. To exclude this possibility, we titrated amounts of K413Q IDH2 enzyme to match the reaction rates of IDH2 WT (Fig. 1d) and analysed the reaction mixtures for metabolite content. Even if we approached 2OG yield similar to IDH2 WT (Fig. 1d), 2HG production was lower after reactions of the K413Q IDH2 mutant (Fig. 1d). We conclude that acetylation of K413 residue also inhibits 2HG production by IDH2.

Subsequently, to analyse 2HG production in the cellular environment, IDH2 variants were overexpressed in 293LTV cells, and metabolites were estimated in cell pellets using GC-MS. Cells overexpressing K413Q IDH2 mutant diminished 2HG production by half when compared to the overexpressed WT IDH2 (Fig. 1e). All these results are consistent with the dual function of acetylated lysine 413 in the inhibition of both oxidative decarboxylation and 2HG production by IDH2.

Because IDH2 reaction is reversible and includes also RC function, we assayed ^13^C metabolic flux to quantify the extent of RC. Measuring RC using *in vitro* assay with purified enzyme starting with NADPH and 2OG is intricate, or virtually impossible, given the inhibition of arising NADP^+^ as concluded by Leonardi *et al*. for IDH1^25^. We, therefore, used ^13^C labeled of 1−^13^C-glutamine, and measured incorporation into citrate (Fig. 1f), which is specific for RC flux. We have observed specific labelling of M+1 citrate, when respective incorporation of heavy carbon into citrate pool was around 4% for both IDH2 WT and IDH2 K413Q, respectively. Since citrate abundance was slightly increased (Fig. 1f) in IDH2 K413Q expressing cells, total pool of heavy citrate is in fact elevated, suggesting that lysine 413 might possess a regulatory function in promoting RC.

### SIRT3 deacetylates IDH2 *in vitro*

**Figure 2:**
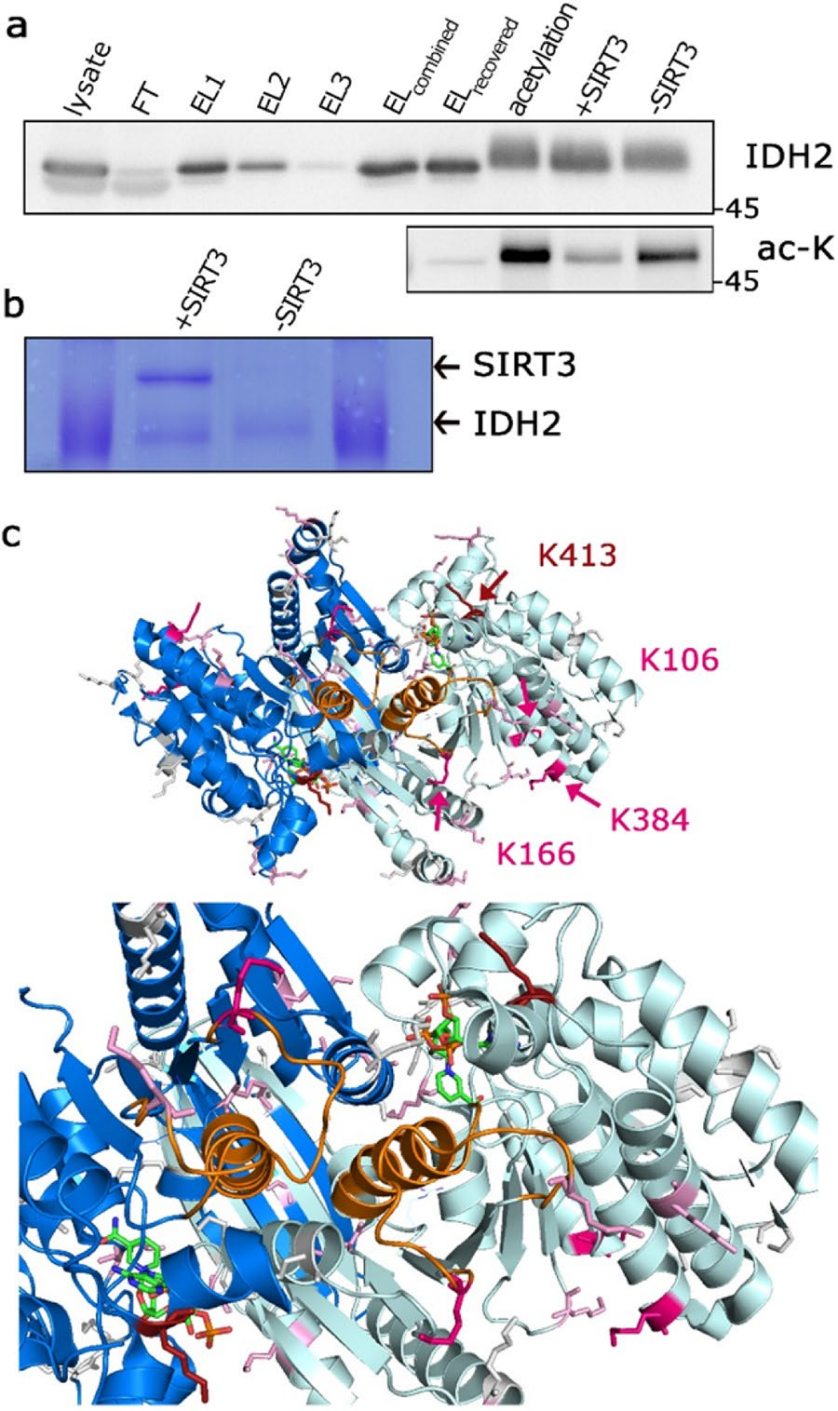
SIRT3 deacetylates IDH2 *in vitro*. **(a)** Western blot analysis of the purified IDH2, indicating that treatment of acetylated IDH2 (“acetylation”) with recombinant SIRT3 deacetylates IDH2, performed with anti-IDH2 and anti-acetyl-lysine antibodies. Western blot was performed using equimolar amount of protein. The lanes contain following fractions: whole cell lysate (*lysate*), flow-through (*FT*), three sequential steps of elutions (*EL1*, *EL2*, *EL3*), combined three elutions (*EL*_*combined*_), following recovery by 10 K Amicon (*EL*_*recovered*_), protein after acetylation step (*acetylation*), protein after treatment with recombinant SIRT3 (+*SIRT3*), and parallel sample without SIRT3 (−*SIRT3*). **(b)** Coomassie blue-stained PAGE of the purified IDH2 used for LC-MS analysis of acetylated lysines with indicated recombinant SIRT3. **C)** Model depicting IDH2 structure with NADPH. The reaction center is depicted in orange, K413 in red, K106, 166, and 384 in magenta, other possibly acetylated/deacetylated lysines in pink.

To evaluate the SIRT3-specificity towards the particular lysine residues within the IDH2 sequence, we artificially acetylated the purified IDH2 *in vitro* using sulfo-NHS acetate (Fig. 2a, Supplementary Fig. S2B). Subsequently, we applied the human recombinant SIRT3 onto acetylated samples, in order to deacetylate IDH2 in the presence of cofactor NAD^+^. Posttranslational modifications of the treated IDH2 samples (Fig. 2b) have been identified by mass spectroscopy (LC-MS), with a focus on acetylated lysines. Confirming that IDH2 is indeed a substrate of SIRT3, we detected deacetylation of IDH2 by SIRT3 using western-blot (Fig. 2a, Supplementary Fig. S2B), and specific deacetylation of the lysines 106, 166, 384, and 413, using LC-MS analysis (Fig. 2c, Supplementary Fig. S2). Deacetylation was not obtained in the presence of SIRT3 inhibitor nicotinamide (NAM, Supplementary Fig. S2). Interestingly, the lysines 106, 166, 384 align the cavity of the reaction center (Fig. 2c), containing the helix α10 (residues 311 to 326) and the loop of the residues 152 to 167^26^. Moreover, acetylated lysines 106, 166, and 384 were detected also in the sample of IDH2 WT purified from untreated cells, although in only low amount (less than 1%, estimated from the intensity ratios of acetylated and non-acetylated peptides, respectively, even though the exact ratio of those peptides cannot be calculated due to the different ionization conditions of acetylated and non-acetylated peptides during MS analysis). Our results suggest that additional lysines of IDH2 located in the proximity of the reaction center are targets of site-specific deacetylation catalysed by SIRT3.

### NADP^+^-driven NADPH formation at overexpressed or silenced SIRT3

**Figure 3:**
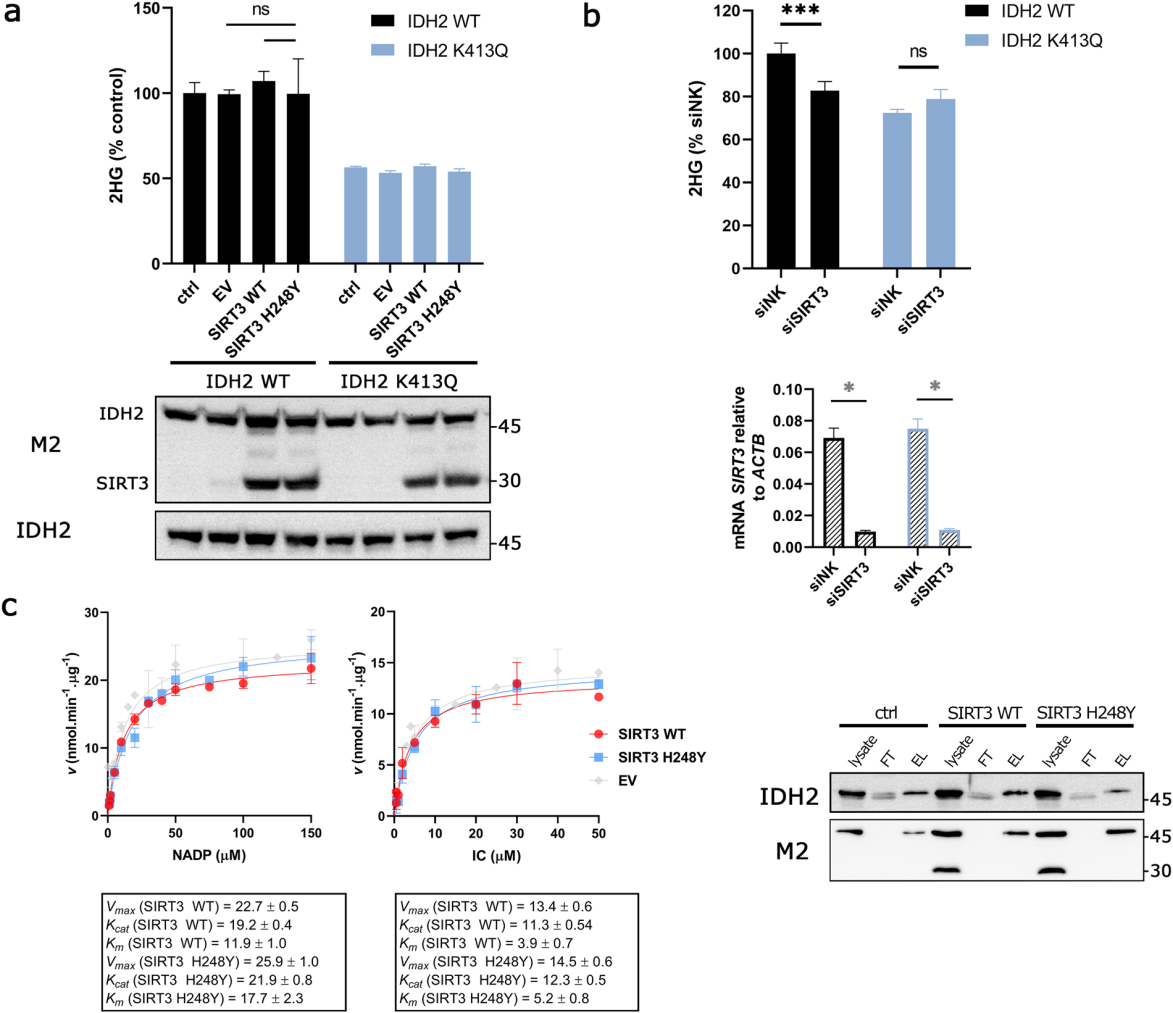
Role of SIRT3 in IDH2 function. **(a)** Cellular 2HG analysed by LC-MS from the control, pcDNA3.1 (EV), SIRT3 WT and SIRT3 H248Y-transfected cells overexpressing IDH2 WT or K413Q, respectively. Underneath there is depicted western blot of the corresponding cell lysates of the analysed samples demonstrating transfection of SIRT3 and H248Y along with IDH2 variants (both detected by anti-M2 antibody). Western blot detecting IDH2 expression in stable cell lines. N = 4. ns p = 0.4608 and p = 0.3353, respectively, calculated using one-way ANOVA Tukey´s multiple comparisons test. **(b)** Cell 2HG analysed by LC-MS from siSIRT3-transfected IDH2 WT and K413Q expressing cells. N > 5. ***p = 0.0001, ns p = 0.6994, calculated using one-way ANOVA Tukey´s multiple comparisons test. The graph below depicts mRNA expression analysis demonstrating silencing of *SIRT3*. N = 2. *p < 0.0001 calculated using the Unpaired t-test. **(c)** Michaelis-Menten plots for IDH2 NADP^+^-driven IDH2-mediated NADPH formation as dependent on concentrations of NADP^+^ and isocitrate transfected with SIRT3 WT and SIRT3 H248Y, respectively. Box contains values of *V*_*max*_ and *K*_*m*_. Unit of *K*_*cat*_ for IDH2 WT/K413Q is *s*^*−1*^; data are expressed as mean ± s.e.m. Western blot depicts the purification of IDH2 from SIRT3 transfected or non-transfected cell lysates. Note that SIRT3 proteins tagged with 3xFLAG were not eluted with FLAG peptide.

To analyse the relevance of SIRT3 specificity for IDH2 in the cellular environment, we further transfected the cell lines expressing IDH2 WT and K413Q by SIRT3 or its inactive mutant H248Y^27^ and analysed cellular metabolites (Fig. 3a). Both SIRT3 variants co-localized with mitochondrial GFP as assayed by confocal microscopy (Supplementary Fig. S3). Interestingly, transfection of SIRT3 WT or H248Y mutant does not result in significant changes in 2HG production in IDH2 WT nor in K413Q-expressing cells. However, such a result could suggest a low acetylation rate, so the effect of SIRT3 would be masked by the lack of acetylated lysines. We, therefore, silenced SIRT3 and measured 2HG levels. The silencing of SIRT3 resulted in approx. 20% decrease of cellular 2HG production in IDH2 WT, but not IDH2 K413Q-expressing cells (Fig. 3b). To further demonstrate the SIRT3 role in IDH2 catalysis, we assayed the IDH2 kinetics of IDH2 purified from cells transfected with WT SIRT3 or inactive H248Y SIRT3 mutant (Fig. 3c). Obtained changes of NADP^+^ and IC-dependent kinetics were not significant in our assay.

### Lack of evidence for GCN5L role in the regulation of IDH2

**Figure 4:**
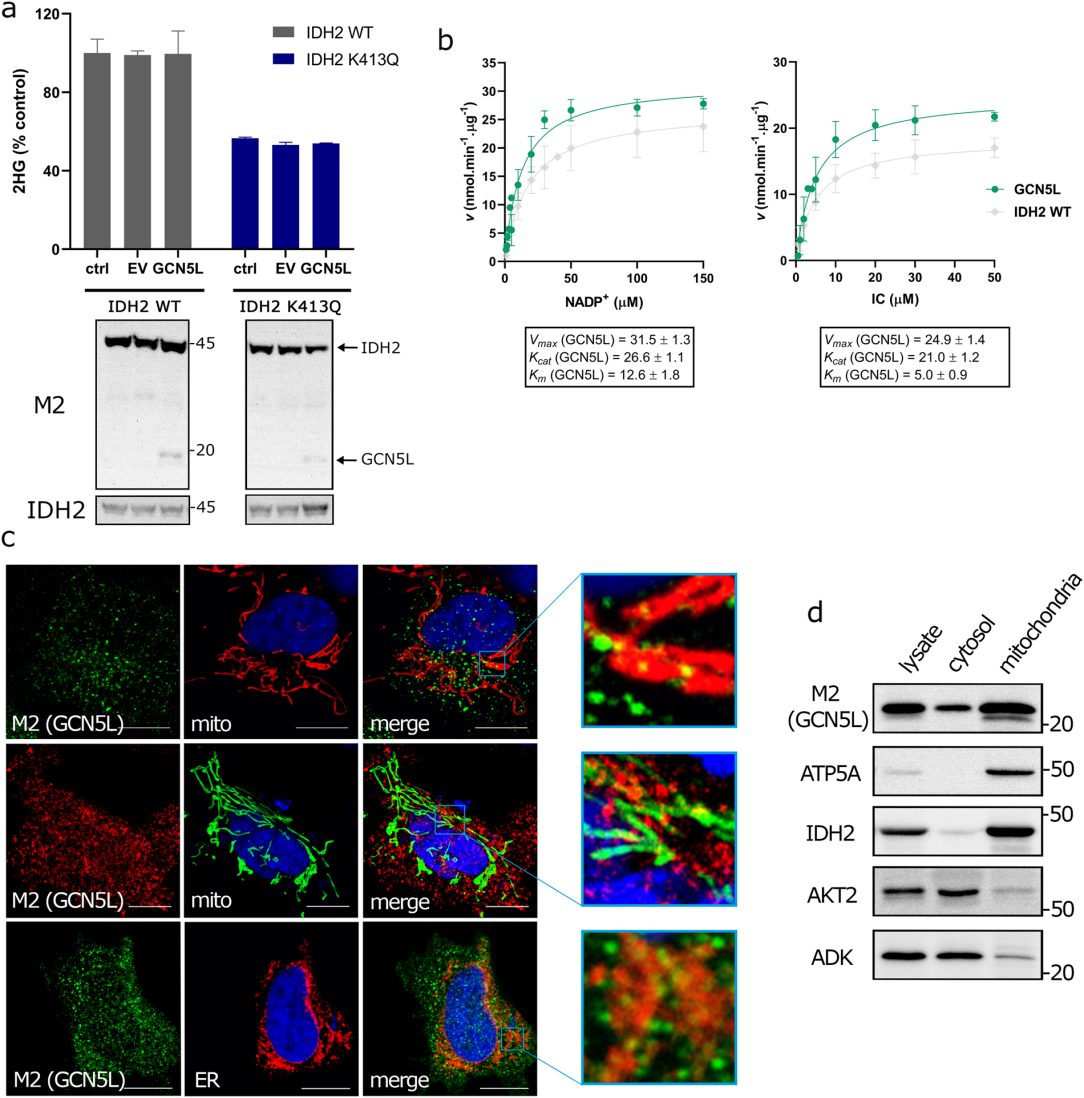
Role of GCN5L in acetylation of mitochondrial IDH2. **(a)** Cellular 2HG analysed by LC-MS from the control (ctrl), pcDNA3.1 (EV), and GCN5L-transfected cells overexpressing IDH2 WT or K413Q, respectively. N > 5. Underneath there is depicted western blot of the corresponding cell lysates of the analysed samples demonstrating transfection of GCN5L along with IDH2 variants (both detected by anti-M2 antibody). Western blot detecting IDH2 expression in stable cell lines. **(b)** Michaelis-Menten plots for NADP^+^-driven IDH2-mediated NADPH formation as dependent on concentrations of NADP^+^ and isocitrate (IC) for purified protein from the IDH2-overexpressing SHSY5Y cells transfected with GCN5L. Box contains values of *V*_*max*_ and *K*_*m*_. Unit of *K*_*cat*_ for IDH2 WT/K413Q is *s*^*-1*^; data are expressed as mean ± s.e.m. **(c)** Confocal microscopy images of GCN5L transfected cells, GCN5L detected by primary anti-M2 antibody and labelled by secondary antibody conjugated with Alexa-Fluor 568 (red) or Alexa-Flour 488 (green); mitochondria-targeted GFP (mito-GFP, green); mitochondria-targeted Keima-Red (red), ER-targeted Tomato fluorescent protein (tdTomato-ER-3, red); overlay of the red and green channels (merge). The scale corresponds to 10 µm. **(d)** Western blot depicting mitochondrial and cytosolic localization of GCN5L (detected by Flag-tag, M2), whole-cell lysate, cytosolic fraction, and mitochondrial fraction.

The mitochondrial GCN5L protein has been previously implicated in protein acetylation^19^. We analysed the possible role of GCN5L-mediated acetylation in relation to IDH2. At first, we transfected IDH2-overexpressing cells with GCN5L and analysed cellular metabolites, including 2HG. Ectopic expression of GCN5L in IDH2-expressing cells does not result in changes in cellular 2HG (Fig. 4a). Moreover, the kinetics of IDH2 was not significantly affected by GCN5L transfection (Fig. 4b, Supplementary Fig. S4); the reaction exhibited similar *K*_*m*_ and *V*_*max*_ for the NADP^+^- and IC-driven NADPH formation (and hence also for the assumed 2OG production). We conclude that GCN5L-mediated acetylation of IDH2 does not inhibit the NADP^+^-dependent IDH2 reaction.

Importantly, cellular localization of GCN5L analysed by confocal microscopy exhibited mostly extra-mitochondrial localization (Fig. 4c). To confirm the localization of GCN5L, we created a cell line stably expressing GCN5L-Flag and performed cell fractionation in order to enrich mitochondrial fraction (Fig. 4d). Cytosolic fraction contained a small amount of IDH2 (mitochondrial matrix marker) and did not contain any α-subunit of mitochondrial ATP-synthase (ATP5A, mitochondrial inner membrane marker) confirming that isolation of mitochondria was performed with rather high purity. A dominant fraction of the AKT2 and adenylate kinase 1 (ADK) was detected in the cytosolic fraction. According to the western blot, a significant portion of GCN5L protein localizes in the cytosol and predominant part in mitochondria (M2, Fig. 4d). Given the substantial extra-mitochondrial localization of GCN5L, we conclude that the lack of effect of GCN5L on mitochondrial IDH2 can be caused by weak mitochondrial localization of the GCN5L acetylase.

### 2HG production by IDH2 in the glucose-free medium is regulated independently of K413 acetylation

**Figure 5:**
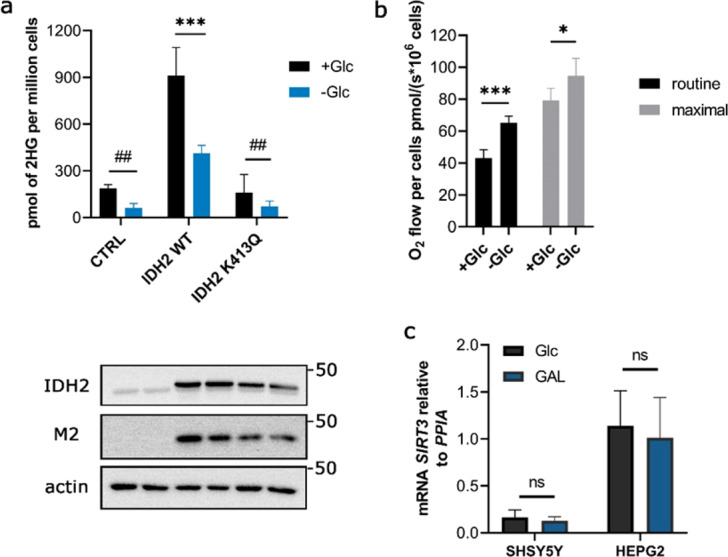
2HG production by IDH2 in the glucose-free medium conditions. **(a)** GC-MS analysis of 2HG levels in glucose-containing (+Glc) and glucose-free medium (−Glc) in control cell line SHSY5Y, or SHSY5Y cells expressing IDH2 WT or K413Q. Below there are depicted respective western blots for IDH2, M2, and actin. N = 3. ***p < 0.0001 using one-way ANOVA, ^##^p < 0.01 (p = 0.0055 and 0.0012, respectively) calculated using Unpaired t-test. **(b**) Respiration rates of SHSY5Y cells cultivated in glucose-containing (+Glc) and glucose-free medium (−Glc) measured by Oroboros 2 K oxygraph; routine respiration refers to endogenous respiration of million cells, maximal respiration refers to maximally uncoupled respiration using the mitochondrial uncoupler FCCP. N > 5. ***p = 0.0006, *p = 0.0150, calculated using one-way ANOVA Tukey’s multiple comparisons test. **(c)** mRNA expression analysis of *SIRT3* in SHSY5Y and HepG2 cells grown in glucose-free medium for 72 hours. N > 4. ns p = 0.3960 and p = 0.6406, respectively, calculated using Unpaired t-test.

We next determined the level of 2HG in glucose-free conditions to address potential changes associated with increased respiration (Fig. 5b) and concomitant NADH/NAD^+^ variation. 2HG was decreased in IDH2 WT expressing cells (Fig. 5a). However, 2HG in cells expressing IDH2 K413Q was also decreased to the same extent, suggesting that acetylation of lysines other than 413 might apply in response to glucose removal. Krebs cycle intermediates were decreased in aglycemia (Supplementary Fig. S5), indicating their higher turnover. Therefore, the reduction of 2HG can be given by favouring the oxidative reaction of IDH2 at high turnover rate of the Krebs cycle. Alternatively, a strong decline of 2HG production might suggest that glucose removal induced robust pro-acetylation conditions in mitochondria, increased NADH production, or decreased expression of SIRT3. However, RT-PCR analysis of SIRT3 in glucose-containing and glucose-free medium did not show any significant changes in *SIRT*3 levels using two independent cell lines (Fig. 5c).

## Discussion

In this study, we aimed to explore the potential for mitochondrial IDH2 to produce 2HG and to clarify its regulation by acetylation and deacetylation. Our motivation to elucidate regulations of IDH2 stems from the facts, that function of IDH2 as a Krebs cycle enzyme might affect both catabolic and anabolic cancer metabolism as well as redox homeostasis, and that 2HG is a potent oncometabolite, so the consequences of its function meet the cancer aetiology and epigenetics issues. We do realize though, that levels of 2HG produced by IDH2 WT in this work are by two orders of magnitude lower than in glioma malignancies harbouring IDH2 oncogenic heterozygous mutations. However, our own unpublished data indicate that 2HG is indeed detectable in the serum of breast cancer patients compared to healthy volunteers (Smolková *et al*., unpublished). In order to investigate basic enzymatic function of IDH2 and its regulations, we use independent cell model with low basal 2HG production, so we studied solely the function of ectopically expressed IDH2 WT, which results in 2–10 times higher 2HG production over background levels (Figs. 1e, 5a). 2HG production is catalysed by a handful of mitochondrial and cytosolic proteins, *i.e*., hydroxyacid-oxoacid-transhydrogenase, glutathione-dependent glyoxylases, phosphoglycerate dehydrogenase, lactate dehydrogenase, and malate dehydrogenase 1/2. Now we present the evidence that IDH2 is capable of 2HG production *in vitro* and that the reduction of 2OG to 2HG is regulated by acetylation/deacetylation dynamics. Our conclusions are based on the several key observations; (*i)* 2HG is detectable in the reaction of the purified IDH2; (*ii)* cellular 2HG level is dependent on IDH2 expression level; (*iii)* acetylation surrogate IDH2 mutant K413Q and knockdown of SIRT3 is associated with lower levels of 2HG.

In general, acetyl-CoA regulates responses to homeostatic perturbations by controlling the equilibrium between anabolic and catabolic reactions. For instance, caloric restriction induces SIRT3^28^, and *vice versa*, the high-fat diet induces expression of the GCN5L acetylase^29^. Besides, hyper-acetylation is linked to various pathologies, such as type 2 diabetes, metabolic syndrome, and cancer. It is estimated that roughly one-third of the mitochondrial proteins are acetylated^30^, although a low level of acetylation is assumed in mitochondria in general (up to 3%)^31,32^. As already mentioned, nonenzymatic acetylation is responsible for the majority of acetylation sites in mitochondria^1,16^ and therefore might be nonregulatory. The assumption of the presented study was that the level of mitochondrial acetylation is constant under our experimental conditions, as our experiments do not disturb the matrix pH and because long-term perturbation is necessary to set the level of acetylation in mitochondria^33^. Therefore, the actual dynamics of acetylation would be exhibited mostly by deacetylation (SIRT3) in an NAD^+^- dependent manner^34^. In an attempt to identify specific sites of IDH2 deacetylation, we used recombinant SIRT3 to deacetylate a pan-acetylated IDH2 and analysed the deacetylated lysines by mass spectroscopy. Yu *et al*.^13^ performed targeted mutagenesis of conserved lysines within the IDH2, *i.e*., lysines 256, 263, 272, and 413, and we do agree that lysine 413 possess the regulatory function. Our data imply three additional lysines to be deacetylated by SIRT3 *in vitro* with high reproducibility (Fig. 2, Supplementary Fig. S2), which deserve further investigation. Moreover, using modeling of the IDH2 structure we mapped the identified deacetylated lysines *in vitro* and found out that lysines 106, 166, and 384 (Fig. 2c) are in the proximity of the essential loop of IDH2 (residues 152 to 167) which interacts with the helix α10 (residues 311 to 326), required for the canonical IDH2 reaction, substrate and Mg^2+^ binding^26^.

We subsequently sought to investigate the effect of acetylation on 2HG production by IDH2. To simulate the effect of acetylation on the function of IDH2 we used acetylation surrogate mutant of IDH2 K413Q which exhibits a substantial decrease of *V*_*max*_ and the shift in apparent affinity for NADP^+^ (Fig. 1b) in accordance to the previous study^13^. While there was reported the inhibition of IDH2 function (oxidative), we provide the evidence for inhibition of 2HG formation by acetylation in the reductive mode of IDH2 (Fig. 1c–e). Analysis of reaction products of IDH2 suggests that 2HG is formed in the IDH2 reaction (following oxidative decarboxylation), although we are aware that the actual stoichiometry of the obtained reaction products (2OG and 2HG) might not reflect the actual situation *in situ*. Moreover, evaluation of the cellular metabolites also showed that the expression of IDH2 K413Q in cells produce half of 2HG compared to IDH2 WT. To use surrogate mutation of acetylated lysines is advantageous for assuming the function of the enzyme *in vitro*; however, further interventions to modulate acetylation status by SIRT3 overexpression in the cells failed to provide a significant difference in the activity of the purified enzyme or 2HG production in cellular environment (Fig. 3a, c). We assume that the intrinsic acetylation of the mitochondrial proteins, including IDH2, was below the level which meets the requirement of SIRT3 activity to deacetylate IDH2. Nevertheless, knockdown of SIRT3 was able to decrease 2HG production in the cells overexpressing IDH2 WT (Fig. 3b). Typically, SIRT3 is activated by elevated NAD^+^ in the range of 280–880 µM^35^. Since the levels of which in mitochondria are around 250 µM and NADH/NAD^+^ ratio of 1:10 is typically established^36^, so the SIRT3 function is plausible. Several physiological situations might affect the NADH/NAD^+^ ratio in mitochondria and activate/inhibit SIRT3 function^37^. For instance, upon the inhibition of Complex I NADH/NAD^+^ ratio rises at the expense of NAD^+^ levels. To analyse conditions of potentially elevated SIRT3 activity, we measured 2HG production in glucose-free culturing conditions and observed profound decrease of 2HG production (Fig. 5a), which was, however, observed also in IDH2 K413Q expressing cells, suggesting that lysine 413 might not be the single regulatory residue which transmits a response to metabolic perturbations within the IDH2. Note, that higher respiration of cells in the glucose-free medium is accompanied by a higher Krebs cycle turnover, documented by a depletion of the respective substrates (Supplementary Fig. S5), which exhausts NAD^+^ pool.

Furthermore, we also address the issue of IDH2-catalyzed RC with respect to acetylation/deacetylation cycle. The role of IDH2 in reductive *vs*. oxidative mode has been discussed mostly for mutant forms of IDH2 where the enzyme IDH2 gains 2HG producing properties and ultimately losses the canonical oxidative function. IDH2 is capable of three modes depending on substrate/cofactor availability. In principle, 2HG produced by IDH2 WT can be derived from glucose; in this case, the oxidative decarboxylation of IC by IDH2 is accompanied by reduction of 2OG into 2HG. If we consider glutaminolysis as a source of carbon for IDH2, 2OG produced by glutaminolysis is split between 2HG (reduction) or IC (RC). The latter is reported in brown fat cells, hypoxia, mitochondrial dysfunction, and breast cancer cells ^9,11,12,38,39^. RC activity might also reflect CO_2_ levels and actual matrix pH. The question remains whether acetylation regulates all three modes of IDH2 functioning, or reductive *vs*. oxidative modes, *i.e*., the reductive NADPH-dependent IC formation. *In vitro* measurement of the RC was reported for IDH1, and the authors concluded that RC is inhibited by NADP^+^ production^25^; therefore, the reaction would require NADPH recycling. Accordingly, we did not succeed in evaluating the activity of purified IDH2 in the reductive mode of the reaction (2OG + NADPH). Employing tracing of glutamine-based carbons incorporated into citrate and malate we estimated RC flux in IDH2 WT and K413Q expressing cells. Given a slight increase of the citrate abundance, but similar relative incorporation of labeled carbon into citrate in two respective cell cultures, we conclude that RC is likely to be affected by K413Q mutation of IDH2, with a low asset, though (Fig. 1F), and therefore the data should be interpreted with caution. We are also unable to review the potential involvement of *K*_*m*_ changes of NADPH or 2OG for IDH2.

Finally, we also attempted to clarify the role of a putative mitochondrial acetylase GCN5L. Unlike SIRT3, which is the exclusive mitochondrial enzyme (Supplementary Fig. 3), our results are in accordance that GCN5L localizes predominantly to extra-mitochondrial space (Fig. 4c, d). Despite possessing the N-terminal mitochondrial targeting sequence as assessed by MITOPROT II (Supplementary Fig. S4), the mitochondrial localization should proceed with targeting probability as low as 34%^40^. Our own data suggest that GCN5L is only partially localized to mitochondria (Fig. 4c,d), although the majority of the signal being concentrated in extra-mitochondrial space, and appears to exhibit punctae-shaped distribution consistent with peroxisome staining. According to an analysis of IDH2 activity and cellular metabolites including 2HG, we presume that expression of GNC5L does not functionally regulate IDH2 WT.

In conclusion, our data describe the mitochondrial production of 2HG as a consequence of mitochondrial IDH2 WT expression and activity, including 2HG production, which is negatively regulated by acetylation of K413 and possibly other lysines. We also provide four newly identified residues in the IDH2 sequence, which are plausible regulatory sites targeted by mitochondrial deacetylase SIRT3.

## Methods

### Materials

We specify all the resources at the specific procedure section. Antibodies used in the study were detecting epitopes of FLAG (M2, Sigma-Aldrich F1804), IDH2 (Abnova H00003418-M01), acetylated-Lysine (Cell Signaling 9441), ATP5A (Abcam ab14748), ADK (Abcam ab138504), AKT2 (SAB 21155), and β-actin (Abcam ab8226).

### Cell cultures

SHSY5Y cells were purchased from ECACC (94030304) and cultured in DMEM containing 4 mM glutamine and 5.5 mM glucose, or 10 mM galactose, respectively. 293LTV cell line was purchased from Cell Biolabs, Inc. (LTV-100) and cultured in DMEM containing 4 mM glutamine and 5 mM glucose. The GCN5L plasmid was a kind gift from Dr. Sack’s laboratory^19^. The IDH2 WT and IDH2 K413Q plasmids were a kind gift from the laboratory of Dr. Denu^13^. Stable cell lines were generated by treatment with G418 (200 µg/ml) after transfection. SIRT3 and SIRT3 H248Y plasmids were purchased from Addgene (#24924, #24917 respectively) and respective ORFs subcloned into the p3XFLAG-CMV14 vector (Sigma-Aldrich). Vector DNA was transfected using Lipofectamine 2000 (ThermoFisher Scientific). For RNA silencing, the predesigned Mission siRNAs (Sigma-Aldrich) were transfected using Lipofectamine RNAiMAX (ThermoFisher Scientific). The decline of RNA was verified using real-time PCR and data calculated using Double Delta Ct analysis.

### IDH2 purification

Cells were lysed in the lysis buffer containing 50 mM Tris-HCl, 100 NaCl, 1 mM EDTA, pH 7.4, 0.1% NP-40, protease inhibitor cocktail, 1 mM PMSF, 100 µM trichostatin A, 10 mM nicotinamide, and overnight incubated with M2 anti-FLAG affinity gel (A2220, Sigma-Aldrich). Protein was eluted using Flag-peptide (F3290, Sigma-Aldrich), diluted by factor of five in the phosphate-buffered saline (PBS) pH 7.4 and recovered by 10 K Amicon filtration. Protein concentration was determined using the BCA assay.

### IDH2 reaction kinetic assay

The IDH2 activity was measured by assessing NADPH fluorescence (excitation 350 nm, emission 450 nm) on a Shimadzu RF 5301PC spectrofluorometer (Shimadzu). The reaction buffer contained 20 mM Tris, 20 mM MgCl_2_, 35 mM NaHCO_3_, pH 8, 100 µM isocitrate or 100 µM NADP, respectively, at 37 °C, varying the initial substrate isocitrate and cofactor NADPH up to 200 µM. Reaction rates were calculated from the initial slope of the NADPH fluorescence (20 s). For subsequent metabolic measurements, reactions were frozen immediately after 120 s. *K*_*m*_ and *V*_*max*_ were calculated from hyperbolic fit in GraphPad Prism 8 assuming the Michaelis-Menten kinetics of reactions. *Kcat* were calculated from *V*_*max*_ values assuming molecular weight of IDH2-Flag 50668.0 g/mol.

### IDH2 structure

The model of human IDH2 depicted in Fig. 2 was created in Pymol Molecular Graphics System, Version 2.0 Schrödinger, LLC. Structure of IDH2 mutant R172K, including NADPH (PDB code 5svn), was used as a template^26^. K172 was changed to R172 by using mutagenesis function within the software a choosing the most suitable rotamer of the amino acid.

### Quantification of cellular metabolites

Quantification of 2HG, 2OG, citrate, or isocitrate from IDH2 assay (Fig. 1C,D) and LTV-293 cells (Fig. 1E) was performed using GC-MS^11^. Internal standard oxalate (10 µl of 1 mg/ml) was added. In case of quantification of ^13^C labeled metabolites internal standard DL-Malic acid-2,3,3-*d*_3_ (10 µl of 100 ug/ml) was added. Samples (cells or reaction mixtures) were extracted with water/methanol/chloroform (1:1:2, w/w/w) and centrifuged (2660 × g, 20 °C, 10 min). In the case of IDH2 assay samples, internal standard *d*_*3*_−2HG disodium salt was used (10 µl of 1 mg/ml). The upper polar phase was transferred into glass vial and lyophilized. The analytes were derivatized with pyridine/*N*,*O*-Bis(trimethylsilyl)acetamide/ chlorotrimethylsilane (4:2:1, v/v/v) at 60 °C for 70 minutes.

Derivatized samples (7 µl) were directly injected in split mode into gas chromatography coupled with mass spectrometry (GC-MS, GC 6890 N, MSD 5973 N, Agilent Technologies, US). The 95% methyl-, 5% phenyl-polysiloxane column (15 m × 250 µm × 0.25 µm) was preconditioned at 100 °C, held 1 minute, then the temperature was increased by 10 °C per minute to 180 °C and held for 1 minute. Finally, the post-column temperature was increased to 300 °C, and this temperature was held for 2 minutes. The mobile phase was helium at 1 ml per minute. Ions were generated by using the electron-ionization mode at 70 eV with the ion source maintained at 230 °C. Ions were measured using SIM acquisition mode. Specific ions have been selected in verified mass spectra of metabolites: oxalate (190.1), citrate and isocitrate (273.2), 2OG (347.3) and 2HG (349.3). Retention times of selected metabolites were confirmed with commercial standards, and detectable specific mass ions were chosen according to their mass spectra. The total time of analysis was 10.4 minutes.

Quantification of the cellular metabolites after transfection of SIRT3, GCN5L, and siSIRT3 (Figs. 3 and 4) was performed at the Department of Metabolomics, IPHYS CAS. The metabolites were extracted using a biphasic solvent system of cold methanol, methyl tert-butyl ether, and water^41^. The bottom (polar) phase was collected, cleaned up using an acetonitrile/isopropanol mixture and after evaporation, the dry extract was resuspended in 5% methanol with 0.2% formic acid followed by separation on an Acquity UPLC HSS T3 column (Waters) using water and methanol both solvents supplemented with formic acid. The metabolites were detected in negative electrospray ion mode (Thermo Q Exactive Plus instrumentation). Isotopically labeled internal standard 2-hydroxyglutaric acid-*d*_*3*_ was used for quantification of 2HG. For other metabolites signal intensities were TIC normalized before statistical analysis.

### *In vitro* acetylation and SIRT3-deacetylation assay

The purified IDH2 was incubated with sulfo-NHS-acetate (70 µg per 1 mg of original protein) for 1 hour at room temperature, inactivated with glycine-TBS (ratio of 4:1 0.1 M glycine and 10**×** TBS, respectively), diluted by a factor of ten in TBS and recovered using 10 K Amicon filters. Deacetylation was performed using the recombinant human SIRT3 (SRP5275, Sigma-Aldrich) in the deacetylation buffer (300 mM NaCl, 2 mM MgCl_2_, 100 mM Tris-HCl, pH 8.0, and 10 mM NAD^+^, or 10 mM nicotinamide, respectively) for 2 hours at room temperature^42^. The products were separated by SDS-page and stained with Coomassie blue for subsequent LC-MS analysis.

### Mass spectroscopy analysis of acetylated lysines

Procedures followed protocols described previously^43^. The SDS-PAGE protein gel stained by Coomassie blue was divided into slices. Gel slices were destained with 25 mM ammonium bicarbonate in 50% acetonitrile at 30 °C for 30 min and dried with 200 μl acetonitrile for 5 min at 30 °C. Dry gel pieces were treated with dithiothreitol (65 °C, 30 min) and iodoacetamide (RT for 30 min in the dark) to reduce and alkylate cysteines. Proteins in gel pieces were digested with 0.1 μg of trypsin/chymotrypsin solution in 50 mM ammonium bicarbonate at 37 °C for 16 h. Peptides were extracted with 50 μl of 2% TFA and 50 μl of 60% acetonitrile. The peptides were dried in the SpeedVac and dissolved in 15 μl of 0.1% formic acid.

The samples were analysed on the UltiMate 3000 RSLCnano system (Dionex) coupled to a TripleTOF 5600 mass spectrometer with a NanoSpray III source (Sciex). The peptides were trapped and desalted with 2% acetonitrile in 0.1% formic acid at flow rate of 5 μL/min on Acclaim PepMap100 column (5 μm, 2 cm × 100 μm ID, ThermoFisher Scientific). Eluted peptides were separated using Acclaim PepMap100 analytical column (3 μm, 25 cm × 75 μm ID, ThermoFisher Scientific). The 70 min elution gradient at the constant flow of 300 nl/min was set to 5% of phase B (0.1% formic acid in 99.9% acetonitrile, phase A 0.1% formic acid) for first 5 min, then with gradient elution by increasing content of acetonitrile. TOF MS mass range was set to 350–1500 m/z, in MS/MS mode the instrument acquired fragmentation spectra with m/z ranging from 100 to 2000.

The resulting raw data were searched against a database consisting of human proteins and common contaminants in Protein Pilot 4.5 (Sciex).

Alternatively, the samples were analysed on the Ultimate 3000 RSLCnano system (Dionex) coupled to an Orbitrap Fusion Lumos Tribrid mass spectrometer with an EASY-Spray source (ThermoFisher Scientific). The peptides were trapped and desalted with 0.1% formic acid at a flow rate of 30 μL/min on C18 PepMap100 μ-Precolumn (5 μm, 5 mm × 300 μm ID, ThermoFisher Scientific). Eluted peptides were separated using EASY-Spray analytical column (3 μm, 15 cm × 75 μm ID, ThermoFisher Scientific). The 65 min elution gradient at the constant flow of 400 nl/min was set to 5% of phase B (0.1% formic acid in 99.9% acetonitrile, phase A 0.1% formic acid) for first 3 min, then with gradient elution by increasing content of acetonitrile. Orbitrap MS mass range was set to 350–2000 m/z, in MS/MS mode the instrument acquired fragmentation spectra with m/z ranging from 100 to 2000.

The resulting raw data were searched against a database consisting of human proteins and common contaminants in Proteome Discoverer 2.2 (ThermoFisher Scientific).

### Statistics

All analyses were performed using GraphPad Prism Ver. 8.0 (GraphPad Software Inc. San Diego, CA). All data were expressed as mean ± standard deviation (s.d.) if not stated otherwise. Statistical comparisons between tested groups were determined using Unpaired t-test or one-way ANOVA with Tukey’s multiple comparisons test, respectively. The specific statistical test used and the specific statistical significance (p value) is indicated in the respective Figure legend.

Details regarding Cell fractionation, Immunocytochemistry, and Real-time PCR are included in the *Supplementary Information* online.

## Supplementary information

Supplementary information.
