# Supplementary material for: SIRT3 and GCN5L regulation of NADP+- and NADPH-driven reactions of mitochondrial isocitrate dehydrogenase IDH2
Source: Sci Rep. 2020 May 26;10:8677. doi: 10.1038/s41598-020-65351-z (PMC7250847; doi:10.1038/s41598-020-65351-z)
Supplement: Supplementary file 1 — Supplementary information. [file 41598_2020_65351_MOESM1_ESM.pdf]

Supplementary Information

SIRT3 and GCN5L regulation of NADP+- and NADPH-driven reactions of mitochondrial isocitrate dehydrogenase IDH2

Katarína Smolková, Jitka Špačková, Klára Gotvaldová, Aleš Dvořák, Alena Křenková, Martin Hubálek, Blanka Holendová, Libor Vítek and Petr Ježek

Supplementary Figure S1

A

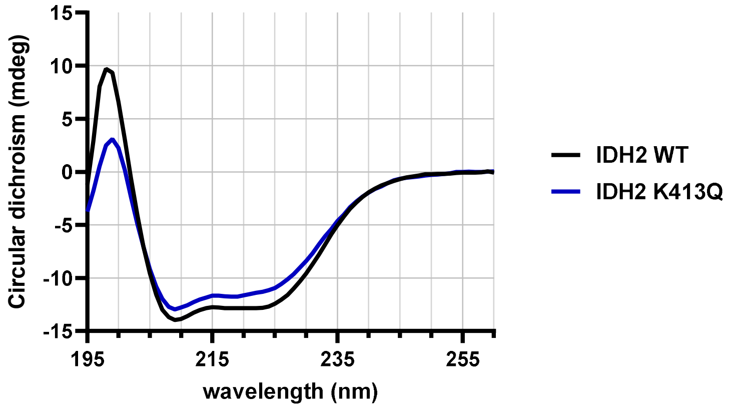

	IDH2 WT	IDH2 K413Q
Helix	70.1 %	63.6 %
Antiparallel	2.5 %	3.5 %
Parallel	2.9 %	3.8 %
Beta-turn	11.3 %	12.7 %
Rndm. coil	13.2 %	16.4 %
Total Sum	**100%**	**100%**

B

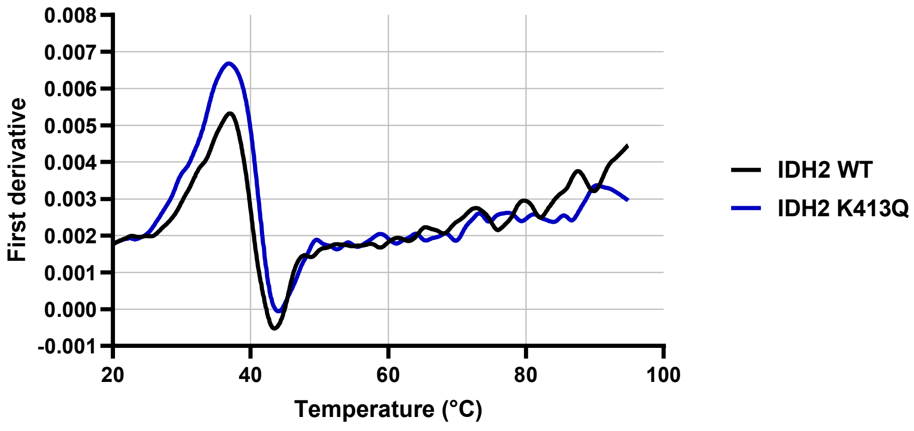

C

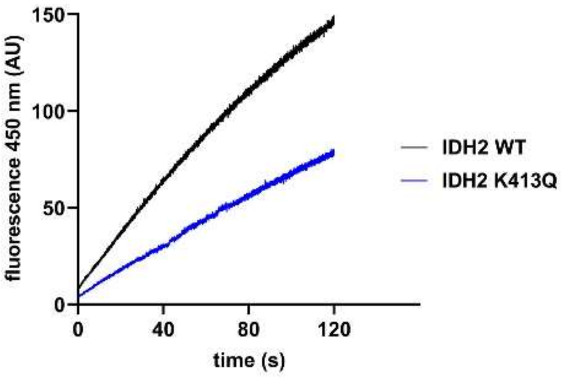

D

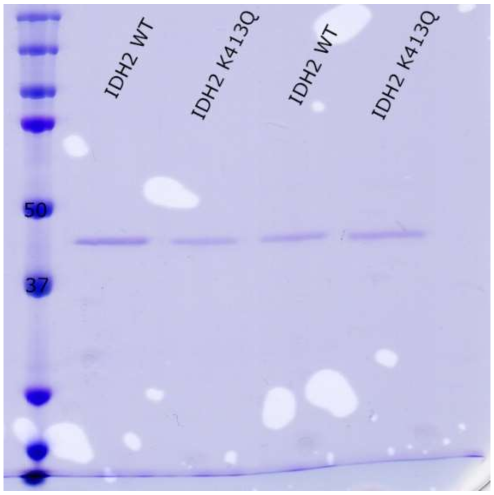

E

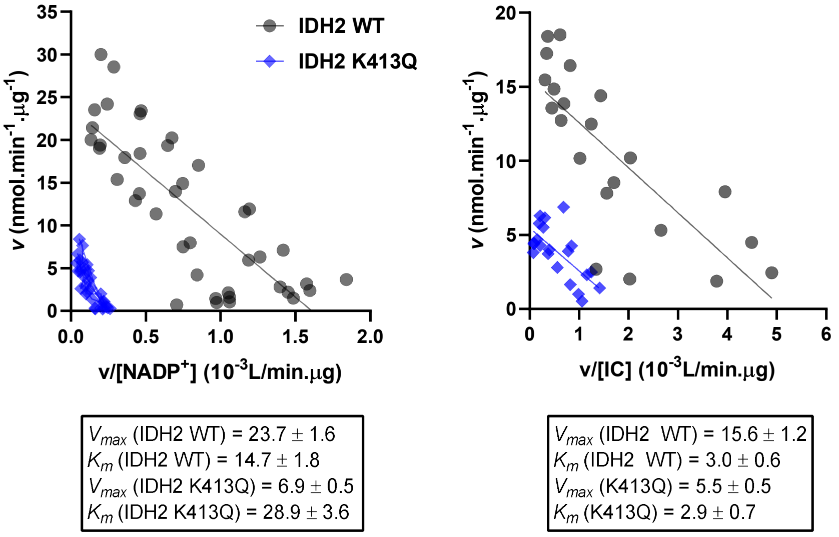

Supplementary Figure S1

A) CD spectroscopy of IDH2 WT and K413Q. *Left*: CD spectra, *right:* calculation of protein secondary structures. **B)** Thermal stability of IDH2 WT and K413Q measured by NanoDSF, melting temperature was 36.5°C (±0.018) and 36.8°C (±0.187) for IDH2WT and K413Q, respectively. **C)** Typical NADPH fluorescence measurement experiment showing the difference of IDH2 WT and K413Q lysates (equivalent amount of the proteins). **D)** SDS gel (10%) demonstrating purification of IDH2 WT and K413Q. Used protein marker is BIO-RAD 161-0375. **E)** Eadie-Hofstee plots for $NADP^+$-driven IDH2-mediated NADPH formation as dependent on concentrations of $NADP^+$ (left) and IC (right). Data are expressed as mean ± s.e.m

Supplementary Figure S2

A

```
MAGYLRVVRSLCRASGSRPA WAPAALTAPTSQEQPRRHYA DKRIKVAKPVVEMDGDEMTR -60
IIWQFIKEKLILPHVDIQLK YFDLGLPNRDQTDDQVTIDS ALATQKYSVAVKCATITPDE -120
ARVEEFKLKKMWKSPNGTIR NILGGTVFREPIICKNIPRL VPGWTKPITIGRHAHGDQYK -180
ATDFVADRAGTFKMVFTPKD GSGVKEWEVYNFPAGGVGMG MYNTDESISGFAHSCFQYAI -240
QKKWPLYMSTKNTILKAYDG RFKDIFQEIFDKHYKTDFDK NKIWYEHRLIDDMVAQVLKS -300
SGGFVWACKNYDGDVQSDIL AQGFGSLGLMTSVLVCPDGK TIEAEAAHGTVTRHYREHQK -360
GRPTSTNPIASIFAWTRGLE HRGKLDGNQDLIRFAQMLEK VCVETVESGAMTKDLAGCIH -420
GLSNVKLNEHFLNTTDFLDT IKSNLDRALGRQKDPDYKDD DDK -463
```

50.667 kDa including Flag-tag
Mitochondrial targeting signal
Amino-acid sequence of IDH2
Linker and FLAG-tag
Lysines targeted by SIRT3
Lysines targeted by SIRT3 with high probablity

B

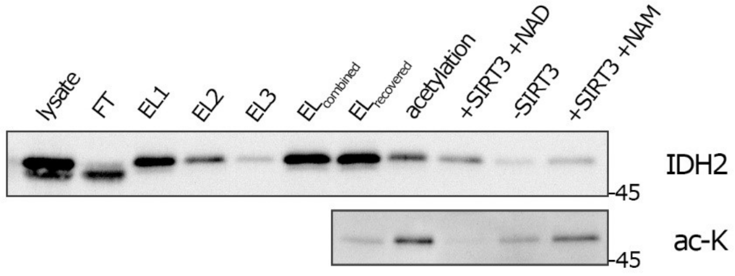

C

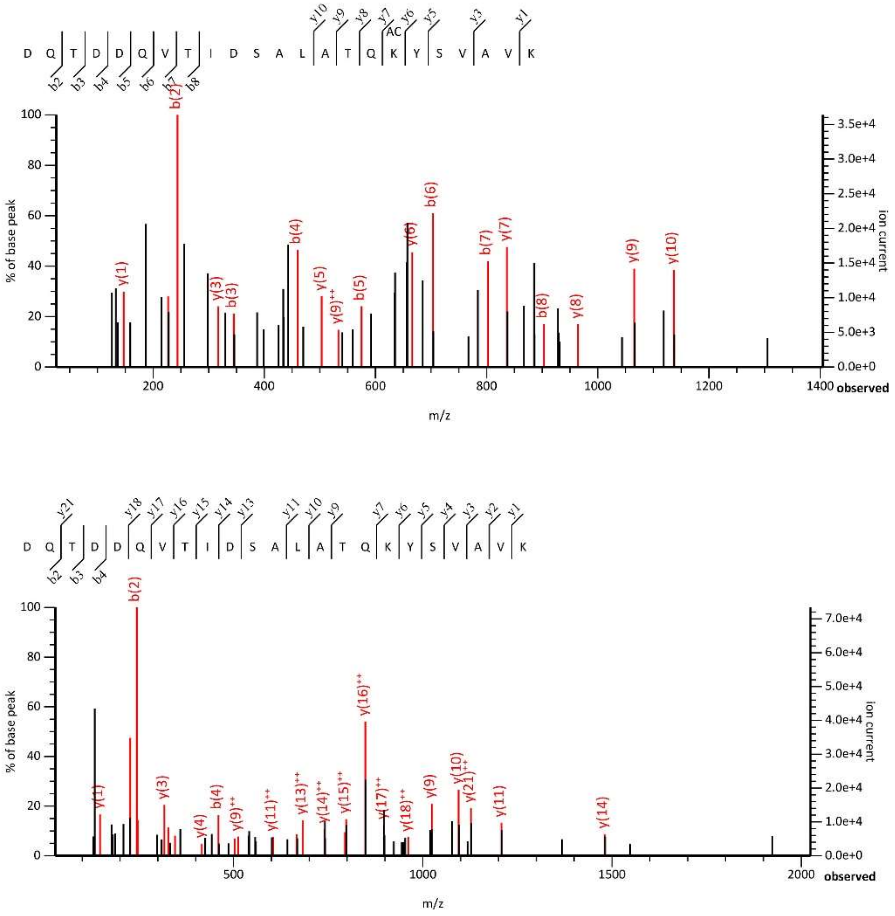
D Q T D D Q V T I D S A L A T Q K Y S V A V K
AC
% of base peak
ion current
m/z
observed
D Q T D D Q V T I D S A L A T Q K Y S V A V K
% of base peak
ion current
m/z
observed

D

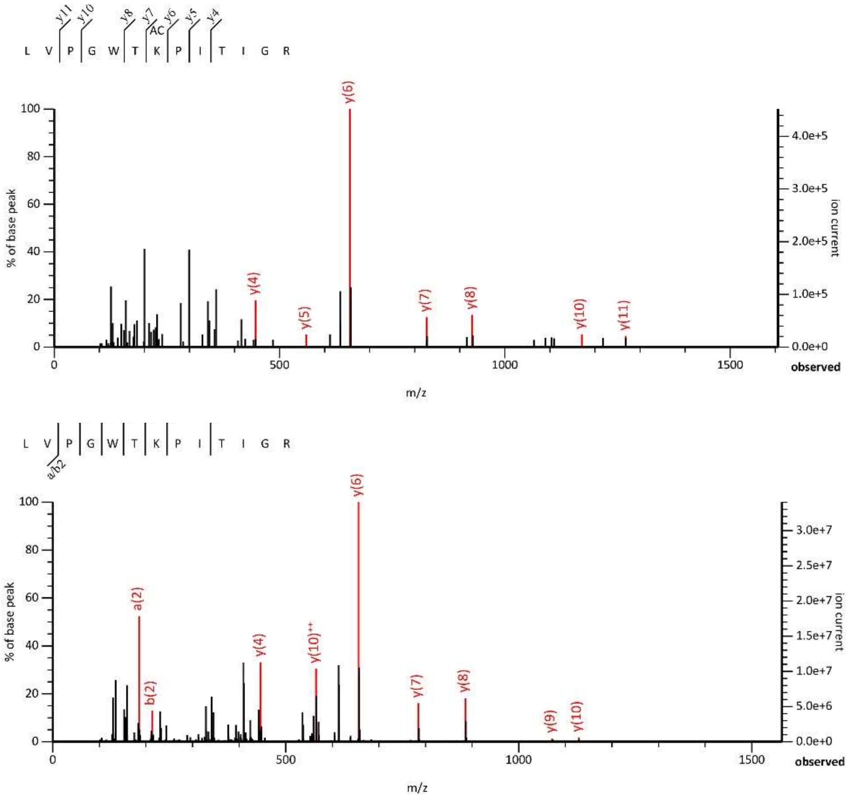
L V P G W T K P I T I G R
AC
% of base peak
ion current
m/z
observed
L V P G W T K P I T I G R
a/b2
% of base peak
ion current
m/z
observed

E

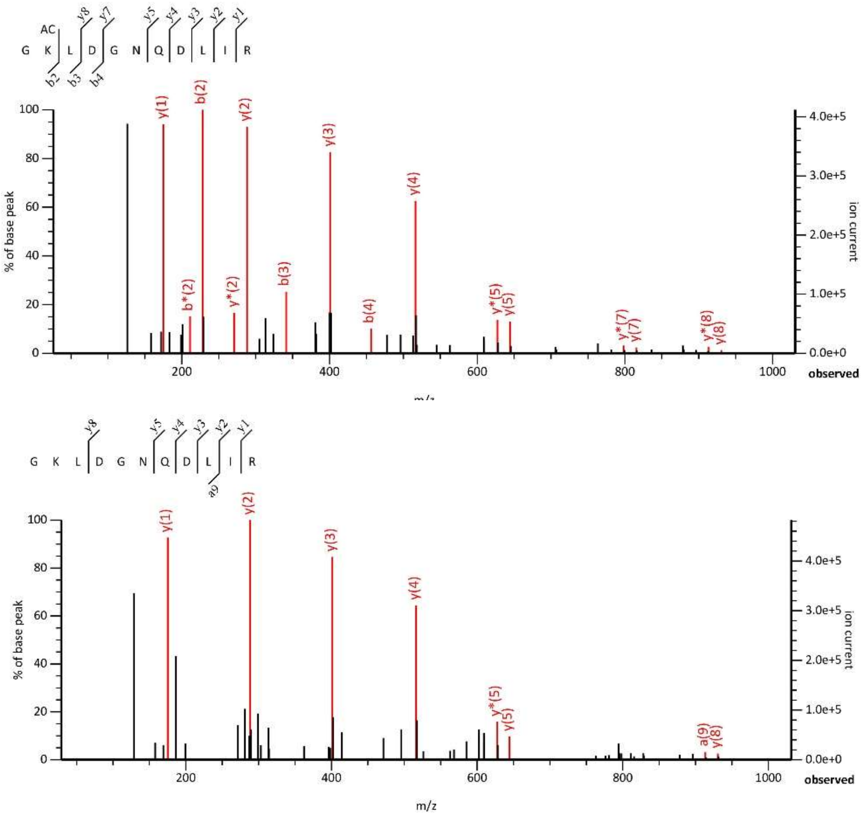
AC
G K L D G N Q D L I R
y8 y7 y5 y4 y3 y2 y1
b2 b3 b4
y(1) b(2) y(2) y(3) y(4) b*(2) y*(2) b(3) b(4) y*(5) y(5) y*(7) y(7) y*(8) y(8)
% of base peak
ion current
m/z
observed
G K L D G N Q D L I R
y8 y5 y4 y3 y2 y1
a9
y(1) y(2) y(3) y(4) y*(5) y(5) a(9) y(8)
% of base peak
ion current
m/z
observed

F

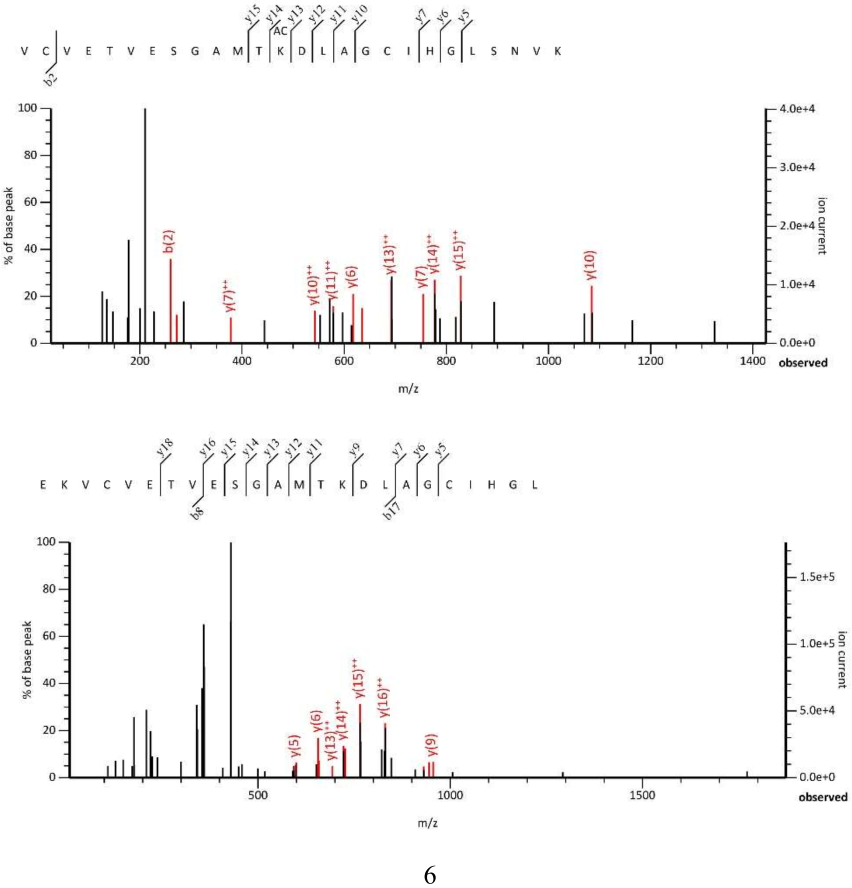
V C V E T V E S G A M T K D L A G C I H G L S N V K
AC
y15 y14 y13 y12 y11 y10 y7 y6 y5
b2
b(2) y(7)++ y(10)++ y(11)++ y(6) y(13)++ y(7) y(14)++ y(15)++ y(10)
% of base peak
ion current
m/z
observed
E K V C V E T V E S G A M T K D L A G C I H G L
y18 y16 y15 y14 y13 y12 y11 y9 y7 y6 y5
b8 b17
y(5) y(6) y(13)++ y(14)++ y(15)++ y(16)++ y(9)
% of base peak
ion current
m/z
observed

G

Annotated Sequence	Modifications	Abundances (Grouped): acetylated sample	Abundances (Grouped): deacetylated sample	# PSMs	Positions in Master Proteins
[R].DQTDDQVTIDSALATQKYSVAVK.[C]			42253.69141	3	P48735 [90-112]
[R].DQTDDQVTIDSALATQKYSVAVK.[C]	1xAcetyl [K17]	370425.0938	114403.4063	2	P48735 [90-112]
[R].GLEHRGKLDGNQDLIR.[F]		7117804.078	1752807.875	8	P48735 [378-393]
[R].GLEHRGKLDGNQDLIR.[F]	1xAcetyl [K7]	12828953.5	315926.5859	4	P48735 [378-393]
[K].NIPRLVPGWTKPITIGR.[H]		3269693.426	4157872.588	58	P48735 [156-172]
[K].NIPRLVPGWTKPITIGR.[H]	1xAcetyl [K11]	13894083	1366632.875	7	P48735 [156-172]
[K].VCVETVESGAMTK.[D]	1xCarbamidomethyl [C2]	29353.3457	407430.5625	3	P48735 [401-413]
[K].VCVETVESGAMTKDLAGCIHGLSNVK.[L]	2xCarbamidomethyl [C2; C18]		100209.2813	3	P48735 [401-426]
[K].VCVETVESGAMTKDLAGCIHGLSNVK.[L]	1xAcetyl [K13]; 2xCarbamidomethyl [C2; C18]	314842.4688	31004.68359	2	P48735 [401-426]

Supplementary Figure S2

A) The protein sequence of IDH2 with MS-identified lysine residues modified by the treatment with recombinant SIRT3 are depicted in green. Lysines targeted by SIRT3 with high probability depicted in magenta. Note that the sequence of IDH2 is a protein sequence identified under UniProt # P48735-1, "Isocitrate dehydrogenase [NADP], mitochondrial." **B)** Western blot analysis of the purified IDH2, indicating that treatment of acetylated IDH2 ("acetylation") with recombinant SIRT3 deacetylates IDH2. The analysis was performed with anti-IDH2 and anti-acetyl-lysine antibodies. Western blot was performed using equimolar amount of protein. The lanes contain following fractions: whole cell lysate (*lysate*), flow-through (*FT*), three sequential steps of elutions (*EL1*, *EL2*, *EL3*), elution fractions (*EL1*, *EL2*, *EL3*) combined ($EL_{combined}$), following recovery by 10K Amicon ($EL_{recovered}$), protein after acetylation step (*acetylation*), protein followed acetylation step (*acetylation*), protein followed treatment with recombinant SIRT3 (+*SIRT3 +NAD*), parallel sample without SIRT3 (-*SIRT3*), and with recombinant SIRT3 and NAM (+*SIRT3 +NAM*). **C) D) E) F)** LC-MS spectra of acetylated and deacetylated peptides, respectively, covering lysine residues 106, 166, 384 and 413, respectively. **G)** Table summarizing abundances of the analyzed peptides.

Supplementary Figure S3

A

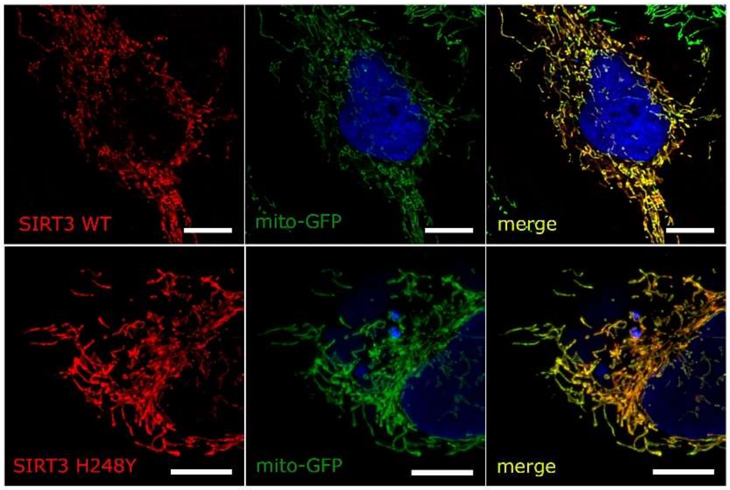

B)

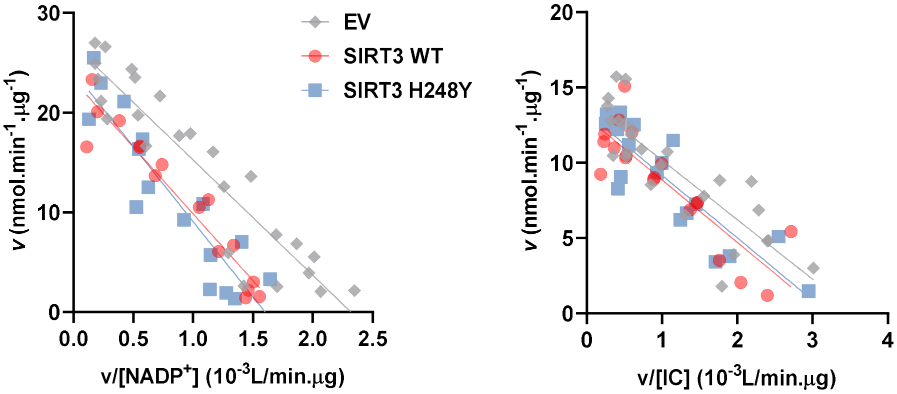

V_{max} (SIRT3 WT) = 23.25 ± 1.1
K_m (SIRT3 WT) = 13.4 ± 1.0
V_{max} (SIRT3 H248Y) = 24.12 ± 1.7
K_m (SIRT3 H248Y) = 15.1 ± 1.8

V_{max} (SIRT3 WT) = 13.01 ± 0.7
K_m (SIRT3 WT) = 4.1 ± 0.6
V_{max} (SIRT3 H248Y) = 13.3 ± 0.7
K_m (SIRT3 H248Y) = 3.1 ± 0.6

Supplementary Figure S3

A) Subcellular localization of SIRT3 WT and H248Y demonstrating mitochondrial targeting, SIRT3 was detected by primary anti-M2 antibody and labeled by secondary antibody conjugated with Alexa-Fluor 568 (red); mitochondria-targeted GFP (mito-GFP, green); overlay of the red and green channels (merge). The scale corresponds to 10 µm. **B)** Eadie-Hofstee plot for IDH2 $NADP^+$-driven IDH2-mediated NADPH formation as dependent on concentrations of $NADP^+$ and isocitrate transfected with SIRT3 WT and SIRT3 H248Y, respectively. Data are expressed as mean ± s.e.m.

Supplementary Figure S4

A

```
MAPGSRGERSSFRSRRGPGVPSPQPDVTMLSRLLKEHQAK -40
QNERKELQEKRRREAITAAT CLTEALVDHLNVGVAQAYM -80
NQRKLDHEVKTLQVQAAQFAKQTGQWIGMVENFNQALKEI -120
GDVENWARSIELDMRTIATALEYVYKGQLQSAPSGSRADY -160
KDHDGDYKDHDIDYKDDDDK

18.47 kDa including Flag-tag
Mitochondrial targeting signal
Amino-acid sequence of GCN5L
Linker and FLAG-xFLAG
```

B

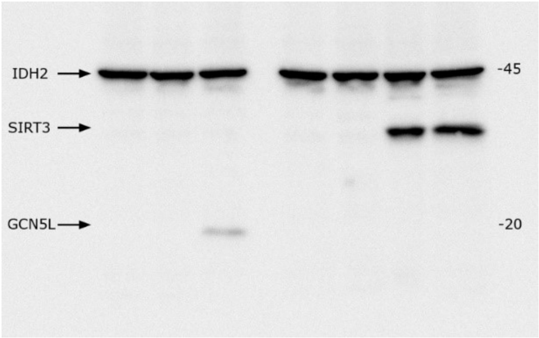

C

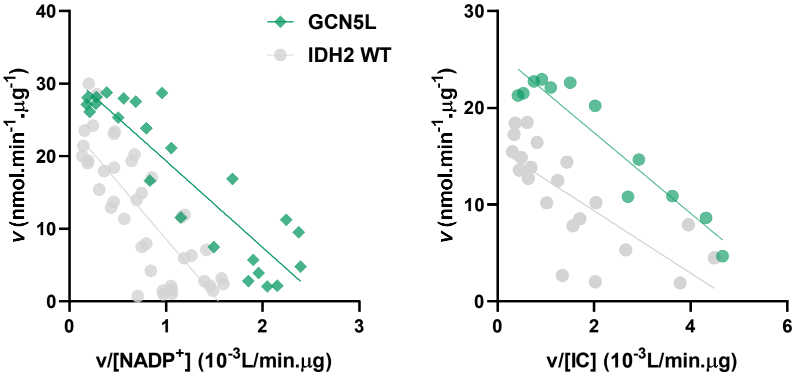

V_{max} (GCN5L) = 31.3 ± 1.7
K_m (GCN5L) = 11.9 ± 1.2

V_{max} (GCN5L) = 25.8 ± 1.2
K_m (GCN5L) = 4.2 ± 0.4

Supplementary Figure S4

A) A sequence of GCN5L used for transfection identified as UniProt # P78537-1 (Uniprot), "Biogenesis of lysosome-related organelles complex 1 subunit 1, BLOC1S1", including sequence features. **B)** Exemplar western blot detecting Flag-tagged proteins IDH2, SIRT3, and GCN5L by anti-M2 antibody. **C)** Eadie-Hofstee plots for $NADP^+$-driven IDH2-mediated NADPH formation as dependent on concentrations of $NADP^+$ and isocitrate (IC) for purified protein from the IDH2-overexpressing SHSY5Y cells transfected with GCN5L. Data are expressed as mean ± s.e.m.

Supplementary Figure S5

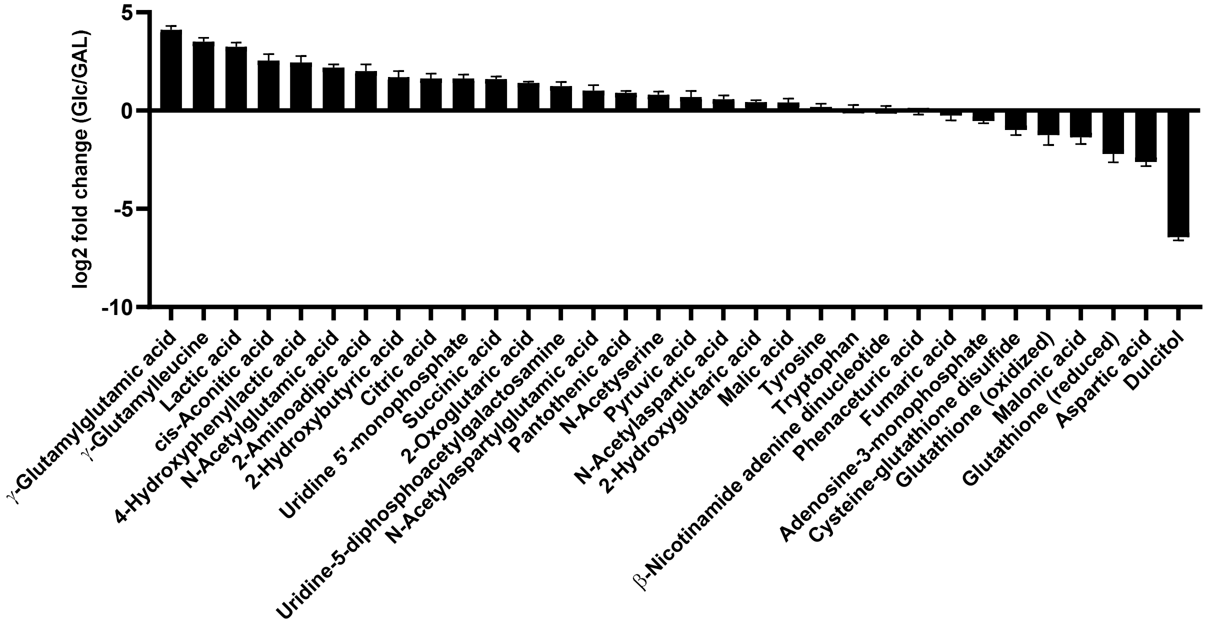

Supplementary Figure S5

Relative abundance of the metabolized analyzed by LC-MS from SHSY5Y cells expressing IDH2 WT cultivated in 5 mM glucose (Glc) or glucose-free medium (-Glc).

Supplementary Figure S6

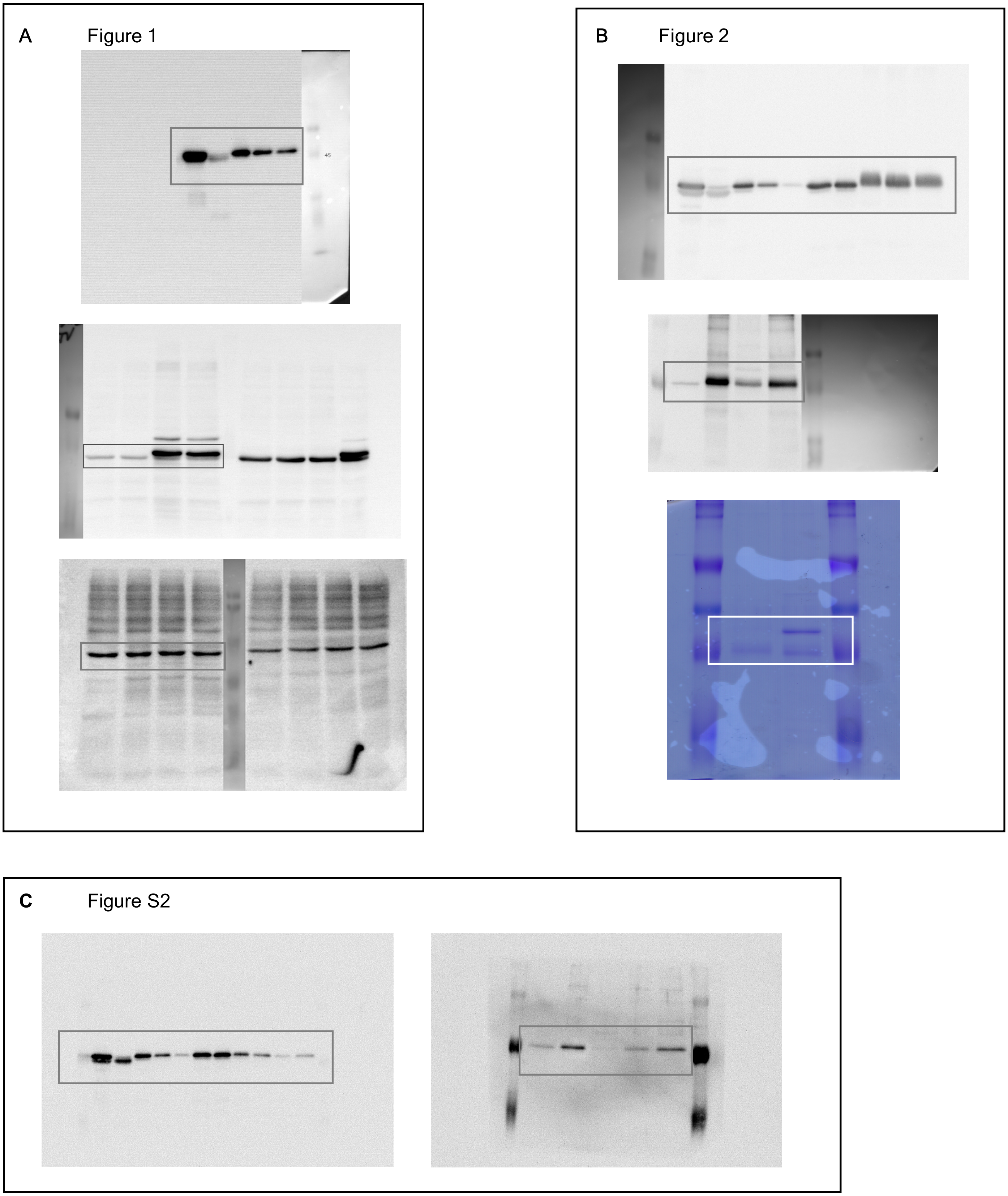

D Figure 3

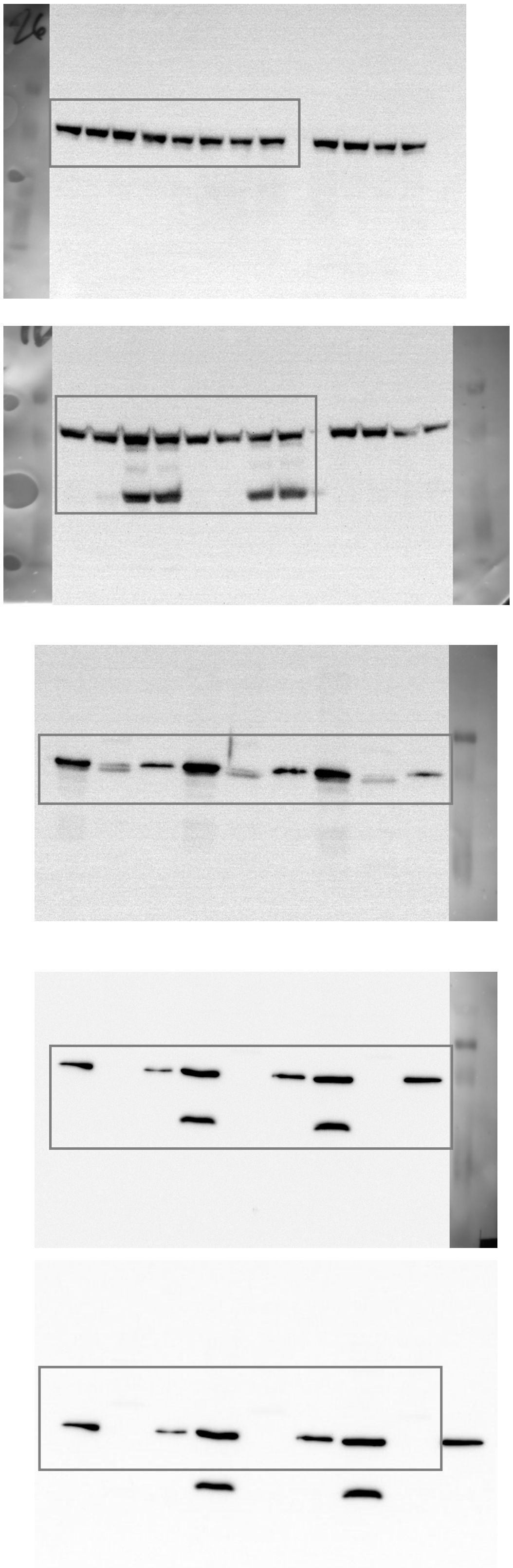

E Figure 4

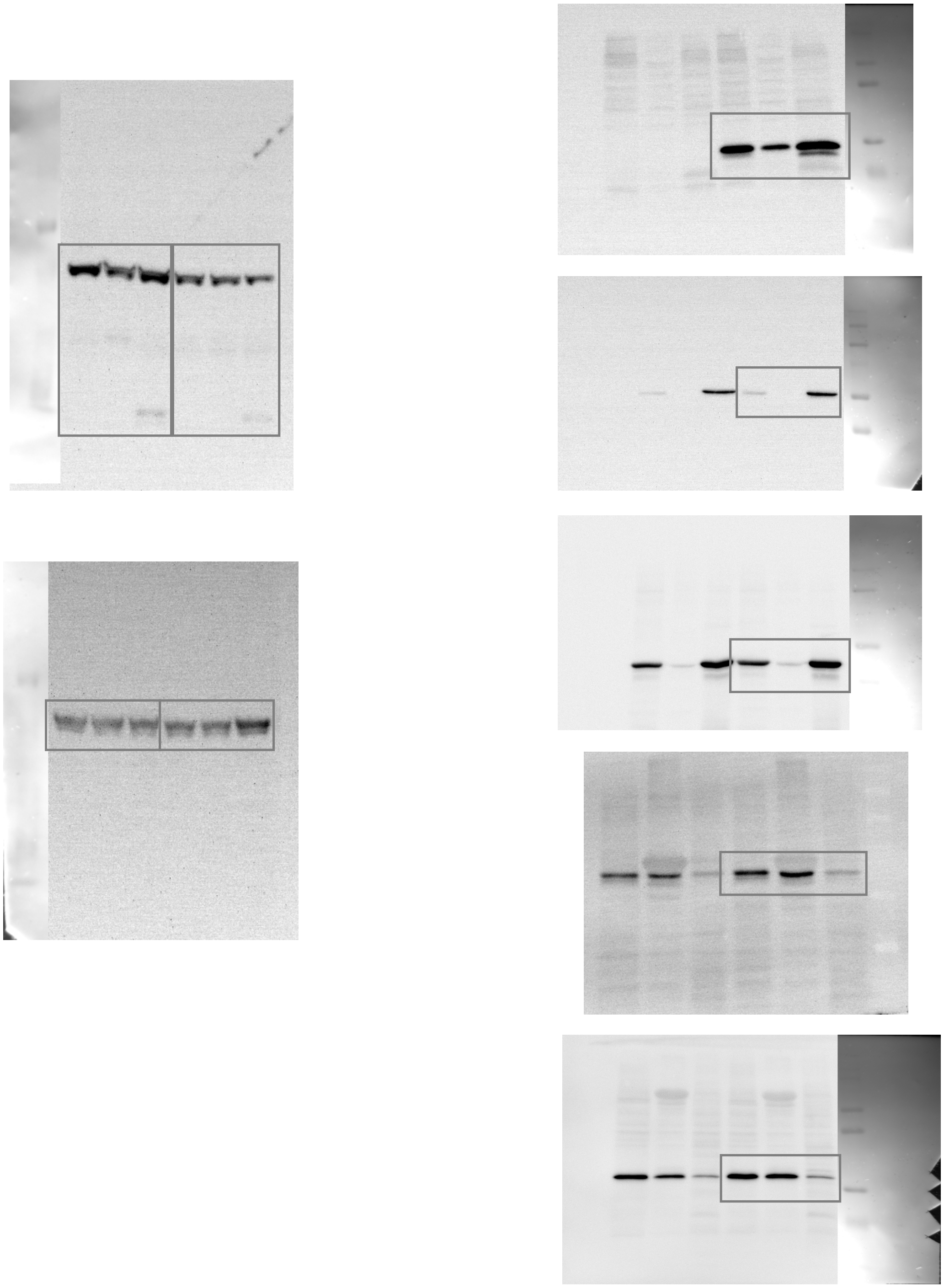

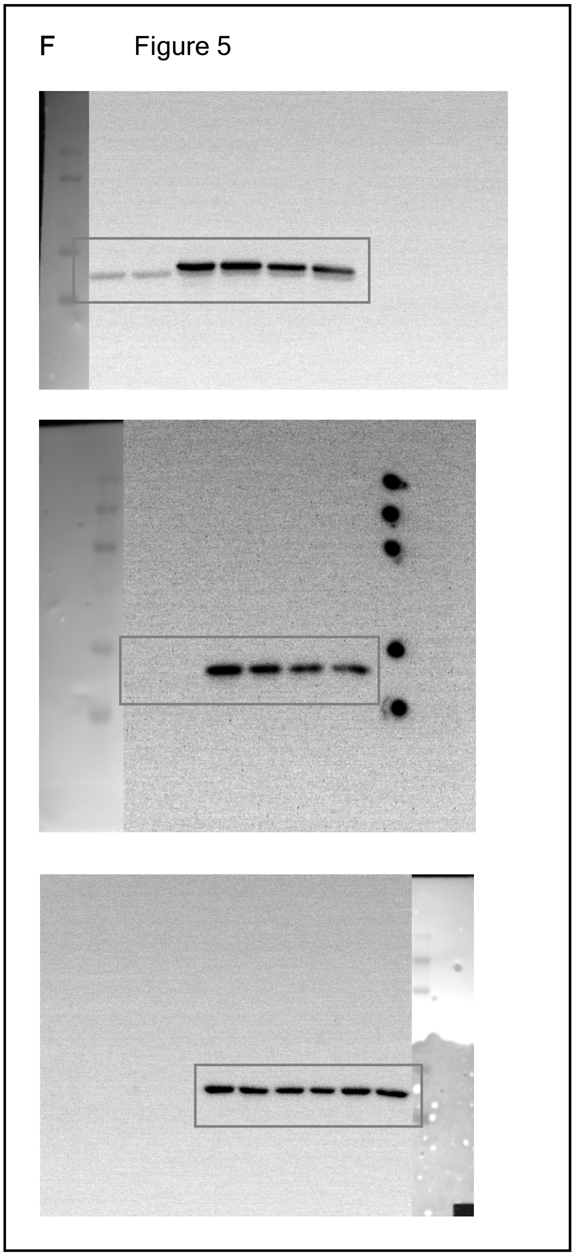

Supplementary Figure S6
The full-length Western blotting and the gel images for the cropped images shown in **A**) Fig. 1; **B)** Fig. 2; **C)** Fig. S2; **D)** Fig. 3; **E)** Fig. 4; **F)** Fig. 5.

Supplementary Methods

Cell fractionation

Cells were harvested from three 150 cm^2 culture dishes by treatment with 0.5% trypsin and washed with PBS. A small part of the cells was lysed in RIPA buffer (lysate) and protein concentration determined. The larger part of cells was resuspended in 2 ml of cold isolation buffer (20 mM HEPES pH 7.2, 1 mM EGTA, 210 mM mannitol, 70 mM sucrose, with 0.1 % BSA and 1 mM PMSF), and homogenized on ice using glass homogenizer. Homogenate was centrifuged 10 min at 1000 × g, 4°C, the supernatant was carefully removed and collected into a new test tube (pellet was discarded) and centrifuged 15 min at 14000 × g, 4°C. The upper part of supernatant (1 ml) was collected again, and the concentration of protein was determined (cytosol). The rest of the supernatant was discarded. Then the pellet (mitochondrial fraction) was washed 4-times with 1.5 ml of cold isolation buffer with BSA and once with 1.5 ml of cold isolation buffer without BSA. Finally, the pellet was resuspended in 1.5 ml of KCl buffer (5 mM EGTA, 180 mM KCl, 1 mM HEPES, pH 7.2), centrifuged and resuspended in 200 µl of isolation buffer without BSA (mitochondria); protein concentration was determined. Western blot was loaded with 50 µg of whole cell lysate, cytosolic, and mitochondrial protein, respectively.

Immunocytochemistry

Cells were plated onto glass coverslips and transfected 24 hours post-plating. 48 hours after transfection the cells were fixed using 4% paraformaldehyde (15 minutes), washed two times with PBS, permeabilized with 0.01% Triton X-100 (5 minutes), blocked by 5% donkey serum (1 hour) and stained with primary (anti-M2, 1 hour) and secondary antibody (Alexa-Fluor 568 or Alexa-Flour 488, Life Technologies, 1 hour). The imaging was performed using a Leica SP8 confocal microscope.

Real-time PCR

mRNA was isolated from cultured cells using the Total RNA Purification Kit (Jena Bioscience) according to specified procedure. cDNA was synthesized using TATAA GrandScript cDNA Synthesis Kit (TATAA Biocenter). Real-time PCR reactions were prepared with Forget-Me-Not EvaGreen qPCR Master Mix (Biotium) and performed using LighCycler 480 Instrument platform (Roche). Primer sequences used for amplification of human genes *SIRT3*, *ACTB*, and

peptidylprolyl isomerase A (*PPIA*) were as follows: hSIRT3_F AGCAGCTCCCAGTTTCTTCT, hSIRT3_R AGCAGCTCCCAGTTTCTTCT, hACTB_F GAGCACAGAGCCTCGCCTT, hACTB_R GTGTAGAGCCGCAGAAGCAG, hPPIA_F GTATAAAAGGGGCGGGAGGC, and hPPIA_R CTGCAAACAGCTCAAAGGAGAC. Data were analyzed using Double Delta Ct analysis.

Biophysical techniques

IDH2 WT and K413Q were purified as described above and diluted in the PBS, pH 7.4. Protein concentration was determined and diluted to 0.1 mg/ml for subsequent analyses.

CD spectra were recorded using Chirascan™-plus (Applied Photophysics) spectrometer in steps of 1 nm over the wavelength range of 195-260 nm. Samples at a concentration of 0.1 mg/ml were placed into 0.1 cm path-length quartz cell to the holder and individual spectra were recorded at room temperature. The CD signal was expressed as the ellipticity and the resulting spectra were buffer-subtracted. We used the CDNN software provided with Chirascan CD spectrometer to analyze the ratio of the secondary structures [23].

Thermal stability measurements were performed by NanoDSF (differential scanning fluorimetry) on Prometheus NT.48 instrument (NanoTemper Technologies), label-free technique based on changes of tryptophan and tyrosine fluorescence. 10 µl of IDH2 WT and K413Q proteins were placed into the capillaries and fluorescence was screened over a temperature range from 25°C to 95°C using a heating gradient of 1.5°C×min^{-1}. Unfolding transition midpoints were determined automatically from the first derivative of the ratio of the fluorescence intensities (F350/F330).
